# CO_2_-driven biosurfactant synthesis by bacteria within CCUS

**DOI:** 10.1007/s00253-026-13761-w

**Published:** 2026-02-25

**Authors:** Amanda Pasinato Napp, William Lautert Dutra, Lovaine Silva Duarte, Eduarda Vargas Abati, Francine Melise dos Santos, Clarissa Lovato Melo

**Affiliations:** Pontifical Catholic University of Rio Grande Do Sul, PUCRS - Institute of Petroleum and Natural Resources, Porto Alegre, 90619900 Brazil

**Keywords:** Biosurfactants, Microbial CO_2_ capture, Anaerobic metabolism, Microbial, CCUS, Circular economy, Bioprocess engineering

## Abstract

**Abstract:**

Microbial CO_2_ capture coupled with biosurfactant production represents a promising strategy for greenhouse gas mitigation and sustainable biomanufacturing. This review examines the metabolic and engineering aspects of microbial carbon capture, focusing on both anaerobic and CO_2_-enriched systems within the Microbial-CCUS framework. The structural diversity, physicochemical properties, and industrial applications of microbial biosurfactants are discussed, along with emerging evidence of anaerobic biosurfactant synthesis linked to CO_2_ metabolism. Advances in genetic and synthetic biology, pathway modularization, and systems-level modeling are reshaping the potential to coordinate CO_2_ fixation with biosurfactant biosynthesis. Integrating artificial intelligence with metabolic engineering may further optimize productivity, scalability, and energy efficiency. Despite technical and economic challenges, the convergence of CO_2_ utilization, biotechnology, and digital innovation offers a transformative route toward circular carbon systems and climate mitigation.

**Key points:**

• *Microbial CO*_*2*_ *capture drives biosurfactant synthesis within Microbial-CCUS systems*.

• *Anaerobic and CO*_*2*_*-enriched cultures unlock new routes for sustainable biomanufacturing.*

• *Synthetic biology links carbon-fixation modules to biosurfactant pathways*.

**Graphical abstract:**

**Figure:**
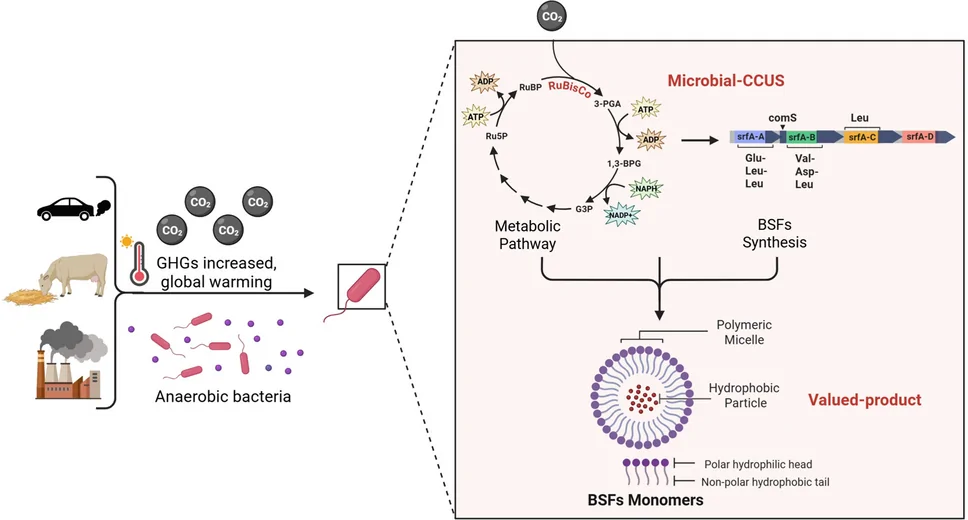

## 1. Introduction

Large-scale anthropogenic emissions of greenhouse gases (GHGs), initiated during the Industrial Revolution, have significantly disrupted Earth’s climate system (Kammerer et al. 2023). Key GHGs, including carbon dioxide (CO_2_), methane (CH_4_), nitrous oxide (N_2_O), hydrofluorocarbons (HFCs), perfluorocarbons (PFCs), and sulfur hexafluoride (SF_6_), absorb and re-emit infrared radiation, enhancing the greenhouse effect and contributing to global warming (Al‐Ghussain 2019; Hanifa et al. 2023). The CO_2_ remains the dominant contributor, rising from historically stable levels below 250 to 420 ppm in 2024, now representing about 76% of atmospheric GHGs (Ritchie et al. 2023). This unprecedented rise is linked to ocean acidification, biodiversity loss, altered precipitation patterns, and threats to food security (Rising et al. 2022).

The Intergovernmental Panel on Climate Change (IPCC) reports a 1.1 °C rise in global temperatures since pre-industrial times, with a projected 0.2 °C increase per decade (Calvin et al. 2023). Moreover, current actions are insufficient to meet the Paris Agreement strategies (Tollefson 2021). The International Energy Agency emphasizes that net-negative emissions are unlikely without large-scale Carbon Capture, Utilization, and Storage (CCUS) deployment (IEA 2021). In this context, conventional CCUS, including amine washing and mineral carbonation, faces economic and scalability constraints, as well as secondary environmental impacts (Hanifa et al. 2023; Thiedemann & Wark 2025).

In contrast, biological CO_2_ sequestration offers sustainable and low-energy alternatives using microorganisms as natural catalysts for carbon fixation (Bardi et al. 2025; Ruan et al. 2025). Microbes employ metabolic pathways such as the Calvin–Benson–Bassham cycle, the Wood–Ljungdahl pathway, and the 3-hydroxypropionate cycle (Duarte et al. 2017; Mistry et al. 2019). These systems not only fix CO_2_ but can generate high-value biomaterials, particularly biosurfactants (Kumar et al. 2018; Sundaram and Thakur 2015). These amphiphilic molecules have broad industrial applications in remediation, cosmetics, agriculture, and pharmaceuticals (Eras-Muñoz et al. 2022; Kugaji et al. 2025; Sarubbo et al. 2022). Their production through microbial CO_2_ assimilation provides a dual advantage, reducing GHG emissions and generating sustainable biomolecules with high added value (Rajkumar et al. 2022). In addition to their role in carbon capture, the contribution of CO_2_-derived biosurfactants to the transition toward greener industries must be demonstrated using quantitative sustainability metrics. Future studies should therefore evaluate microbial-CCUS platforms through indicators such as net CO_2_ balance (kg CO_2_-eq captured or avoided per kg product), energy demand and aeration intensity, carbon utilization efficiency, and life-cycle GHG emissions in direct comparison with petrochemical surfactants and conventional CCUS technologies. Additional parameters, including water and nutrient intensity, biodegradability, ecotoxicity, and biosurfactant performance indicators, will be essential to demonstrate whether microbial-CCUS systems provide genuine environmental advantages, rather than simply transferring impacts between process steps. Integrating its development into sustainability certifications and circular economy frameworks will not only strengthen economic viability but also accelerate the widespread adoption of microbial-CCUS technologies (Kugaji et al. 2025; Sharma et al. 2025).

This review examines anaerobic microorganisms as key agents in CO_2_ fixation and biosurfactant production, highlighting their metabolic pathways, microbial diversity, and recent industrial applications. We further synthesize advances in genetic and metabolic engineering, as well as emerging AI-assisted approaches to optimize yields. By examining technical, economic, and environmental challenges along with scalability and regulatory frameworks, we position anaerobic microbial systems as next-generation platforms that integrate carbon management with sustainable biotechnology.

## 2. CO_2_ capture technologies

**Fig. 1:**
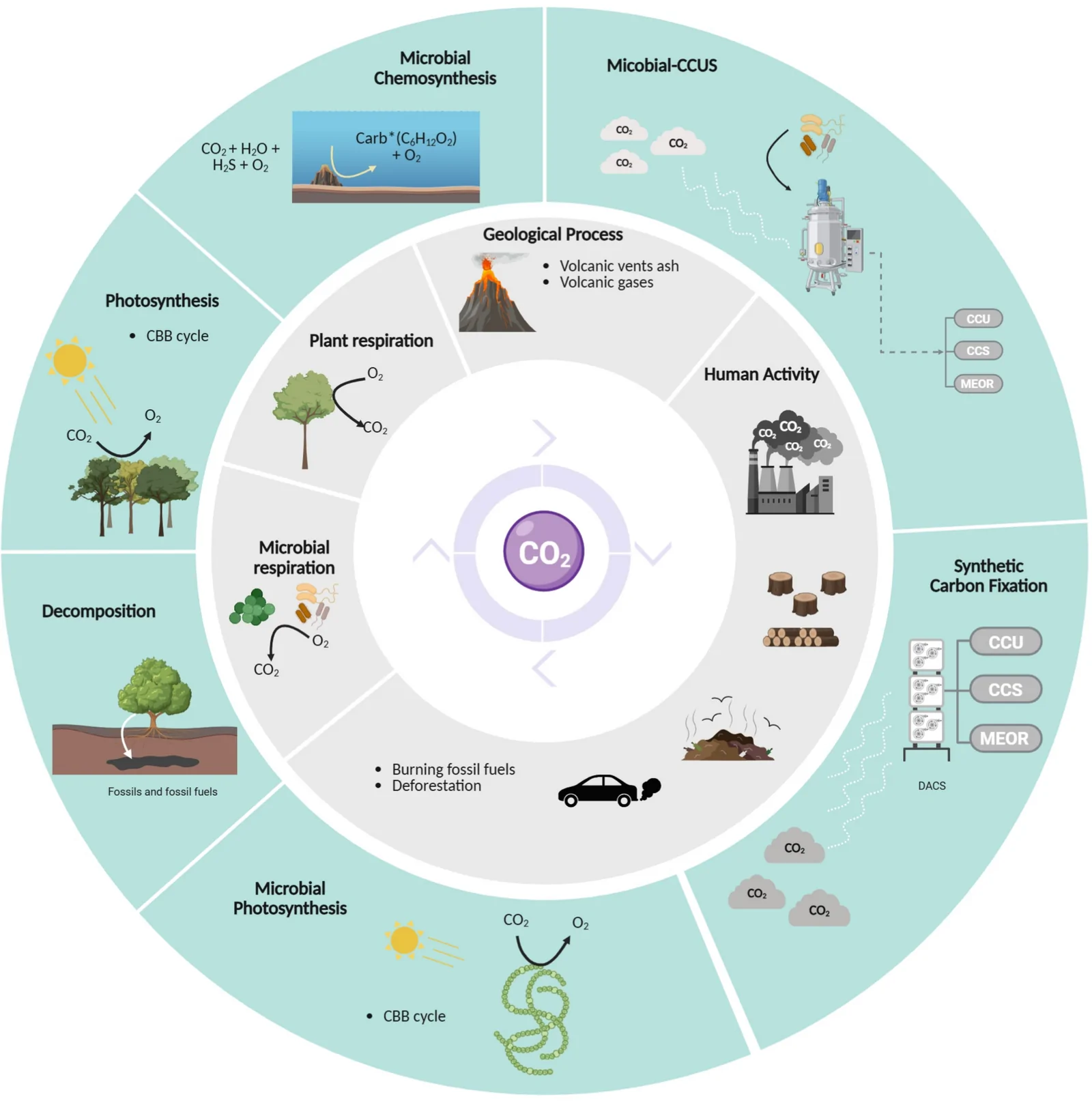
**Global cycle of** **CO**_**2**_ **production and fixation**. The figure illustrates the dual role of CO_2_ as a central chemical compound in metabolic, industrial, and environmental processes. The inner sections depict CO_2_-releasing processes, including anthropogenic activities such as fossil fuel combustion, industrial operations, and deforestation, which are the primary drivers of climate change. Geological processes, including volcanic activity and tectonic emissions, also contribute to atmospheric CO_2_. Plant respiration and microbial respiration release CO_2_ as by-products of aerobic metabolism and organic matter decomposition, respectively. The outer ring represents pathways for CO_2_ fixation. Natural mechanisms include photosynthesis and microbial chemosynthesis, while artificial approaches involve synthetic and synthetic biological carbon fixation technologies, including CCU, CCS, and MEOR. These engineered approaches are increasingly critical for advancing carbon sequestration and mitigating climate change

The global carbon cycle includes interconnected natural and anthropogenic processes that regulate CO_2_ exchanges among the atmosphere, biosphere, and lithosphere (Fig. 1). Natural pathways such as photosynthesis and chemosynthesis operate as biological sinks. In contrast, plant and microbial respiration, decomposition, and geological activity (e.g., volcanic emissions) constitute major sources of atmospheric CO_2_. These biogeochemical mechanisms maintain atmospheric CO_2_ balance but are increasingly disrupted by anthropogenic emissions from fossil fuel combustion, industrial processes, and deforestation (Ángeles et al. 2021).

In response to rising emissions, several CO_2_ capture and storage (CCS) technologies based on engineered physical and chemical principles have been developed to mitigate point-source pollution (Yousaf et al. 2022). CCS systems isolate CO_2_ from flue gases or process streams through post-combustion, pre-combustion, or oxyfuel methods, followed by compression and injection into deep geological formations such as saline aquifers or depleted reservoirs (Karimi et al. 2023).

An evolution of this principle, Direct Air Carbon Capture and Storage (DACCS), removes CO_2_ directly from ambient air using solid sorbents or liquid solvents, offering potential for diffuse and hard-to-abate emissions (Brazzola et al. 2025; Cobo et al. 2022). Similarly, Bioenergy with Carbon Capture and Storage (BECCS) integrates biomass-based energy generation with CO_2_ capture and storage, simultaneously producing renewable energy and achieving net negative emissions (Ataeian et al. 2019; Oh et al. 2025).

Beyond storage-based approaches, Carbon Capture and Utilization (CCU) and Carbon Capture, Utilization, and Storage (CCUS) expand carbon management into industrial and circular pathways. CCU technologies convert captured CO_2_ into fuels, polymers, chemicals, or construction materials, displacing fossil-derived feedstocks and promoting a circular carbon economy. CCUS combines utilization and long-term storage, linking industrial valorization with permanent sequestration and creating an economic bridge toward large-scale mitigation (Cuéllar-Franca & Azapagic 2015; Khandelwal et al. 2021; Rajput & Keshavkant 2025; Ruan et al. 2025). A concise summary of the main carbon management pathways discussed in this section is provided in Box 1.

In parallel, Microbial-CCUS represents an emerging biological strategy that complements engineered systems. Through autotrophic pathways such as the CBB and Wood–Ljungdahl cycles, microorganisms can fix CO_2_ into valuable biomolecules including biosurfactants, bioplastics, and biofuels. This biological capture approach is characterized by low energy demand, scalability, and compatibility with waste gas streams, aligning biotechnological innovation with sustainable carbon management.

Despite their potential, the widespread deployment of CCS, DACCS, BECCS, and CCUS faces economic, technical, and environmental challenges. High energy and water demand, solvent degradation, corrosion, leakage risks, and material limitations remain key obstacles (Ángeles et al., 2021a; Oh et al. 2025). Overcoming these barriers requires continued research to develop cost-effective, durable, and scalable systems. The integration of engineered technologies with natural carbon sinks and biological approaches is essential for advancing sustainable and resilient carbon management.

**Table:** 

**Box 1. Glossary of carbon management pathways**
**CCS:** Capture of CO_2_ from large point sources (e.g., power plants, industries), followed by permanent storage in geological reservoirs (saline aquifers, depleted oil and gas fields). **DACCS:** Direct removal of atmospheric CO_2_ using chemical sorbents or solvents, coupled with geological sequestration. **BECCS:** Combines biomass-based energy production with CO2 capture and storage, achieving simultaneous low-carbon energy generation and removal of biogenic CO2 from the atmosphere. **CCUS:** Integration of CO2 utilization and permanent storage, bridging economic and mitigation goals. **CCU:** Conversion of captured CO2 into industrial products (fuels, chemicals, or materials), promoting circular carbon use. **Microbial-CCUS:** Biological fixation or assimilation of CO2 by microorganisms (e.g., CBB, Wood–Ljungdahl cycles), producing high-value biochemicals.

## 3. Biological CO_2_ fixation

Microbial CO_2_ fixation represents a frontier in carbon capture research, providing solutions to the limitations of conventional CCS and chemical methods. Microorganisms employ diverse biochemical pathways and enzymatic strategies to convert CO_2_ into biomass or value-added compounds, often with greater sustainability and adaptability (Choi et al. 2022; Correa et al. 2025; de Oliveira Maciel et al. 2022).

These strategies are broadly classified into photosynthetic and non-photosynthetic systems (Table 1). Photoautotrophs, including algae and cyanobacteria, rely on light-driven energy conversion, whereas chemoautotrophs and anaerobic archaea exploit chemical energy from inorganic substrates such as hydrogen, sulfur compounds, or ammonia. Although heterotrophs are not primary CO_2_ fixers, some exhibit ancillary pathways that contribute marginally to carbon assimilation (Gong et al. 2018; Maheshwari et al. 2022). Together, these organisms demonstrate the ecological and metabolic diversity of microbial carbon fixation and its biotechnological potential.

**Table 1:** Summary of key microbial CO_2_ fixation pathways

Pathway	Microorganisms	Substrate	**Key Enzymes**	Products	Key Reactions	Environment	References
CBB cycle	Plants, microalgae, cyanobacteria, chemolithotrophic bacteria	3 CO_2 _9 ATP 6 NADPH	RuBisCO	G3P	CO_2_ + RuBP → 3-PGA3-PGA + ATP → 1,3-BPG + ADP 1,3-BPG + NADPH → G3P + NADP + PiG3P + ATP → RuBP + ADP	Photosynthetic environments, terrestrial and aquatic	(Bassham et al., 1954)
rTCA cycle	*Chlorobium limicola*, Proteobacteria, acetogens, sulfur bacteria	2 CO_2 _2 ATP 4 NADPH	2-oxoglutarate synthase, Isocitrate dehydrogenase	Acetyl-CoA	CO_2_ + H_2_O → 2-oxoglutarateCitrate → acetyl-CoA + oxaloacetate	Deep-sea vents, anoxic environments	(Evans et al. 1966)
Wood-Ljungdahl	Acetogens (*Clostridium thermoaceticum*), methanogens	2 CO_2 _1 ATP 4 NADPH	Formate dehydrogenase, CODH	Acetyl-CoA	CO_2_ → formate → formyl-THF → methyl-THF + CO + CoA → acetyl-CoA	Anaerobic environments	(Ljungdhal, 1986)
3HP bicycle	*Chloroflexus aurantiacus, Chloroflexi*chemoautotrophic bacteria, archaea	3 HCO_3_⁻ 5 ATP 5 NADPH	Acetyl-CoA carboxylase, Propionyl-CoA carboxylase	Pyruvate	Acetyl-CoA or Propionyl-CoA + HCO_3_⁻ + ATP → malonyl-CoA or Methylmalonyl-CoA +ADP + Pi → Beta-Methylmalyl-CoA → Pyruvate + Other Intermediates	Aerobic conditions, nutrient-limited habitats	(Strauss and Fuchs, 1993)
3HP/4HB cycle	*Metallosphaera sedula, Acidianus infernus*	2 HCO_3_⁻ 4 ATP 4 NADPH	Acetyl-CoA-Propionyl-CoA carboxylase	Acetyl-CoA	CO_2_ + Acetyl-CoA + ATP → malonyl-CoA + ADP + Pi → 3HP + CoA → Propionyl-CoA + CO_2_ → 4HB → Acetyl-CoA	Anaerobic, acidic, thermophilic environments	(Berg et al., 2007)
Di/4HB cycle	*Ignicoccus hospitalis, Thermoproteus neutrophilus*	1 CO_2 _1 HCO_3_⁻ 3 ATP 4 NADPH	Pyruvate synthase, PEP carboxylase	Acetyl-CoA	CO_2_ + Acetyl-CoA + ATP → malonyl-CoA + ADP + PiSuccinyl-CoA + H_2_ → 4-HB → Acetyl-CoA + H_2_O/Pyruvate + CO_2_ + ATP → Oxaloacetate + ADP + Pi	Geothermal, hydrothermal vents	(Huber et al., 2008)

### 3.1 Photosynthetic CO_2_ fixation

Photosynthesis remains the dominant natural mechanism for CO_2_ assimilation. In photoautotrophic organisms (plants, algae, cyanobacteria), light-dependent reactions generate ATP and NADPH, which power the Calvin–Benson–Bassham (CBB) cycle (Table 1) (Bassham et al. 1954; Karishma et al. 2024). The ribulose-1,5-bifosfato carboxilase/oxygenase (RuBisCO) catalyzes CO_2_ fixation into ribulose-1,5-bisphosphate, producing 3-phosphoglycerate and eventually carbohydrates (Kajla et al. 2022; Li et al. 2024; Liu et al. 2024).

The main photoautotrophic microbial chassis explored for biotechnological CO₂ capture include cyanobacteria such as *Synechocystis* sp. PCC 6803, *Synechococcus elongatus* PCC 7942, and *Nostoc* spp., as well as microalgae such as *Chlorella*, *Botryococcus*, and *Arthrospira* (*Spirulina*), which are widely used as model systems to investigate and rationally engineer photosynthetic carbon fixation pathways (Angermayr et al. 2009; Della Valle et al. 2024; Ighalo et al. 2022; Mills et al. 2020; Mohapatra et al. 2022; Rajkumar et al. 2022).

Microalgae such as *Chlorella* and *Botryococcus* employ Carbon Concentration Mechanisms (CCMs), actively transporting bicarbonate (HCO_3_⁻) and compartmentalizing it in pyrenoids to enhance RuBisCO efficiency (Duarte et al. 2017). Cyanobacteria use analogous carboxysomes. These adaptations improve carbon capture under limiting conditions (Ataeian et al. 2019; Correa et al. 2025; Duarte et al. 2017). Microalgae stand out when compared to terrestrial plants in CO_2_ sequestration due to their rapid growth rates, high photosynthetic efficiency, and ability to utilize concentrated CO_2_ sources such as flue gases. Unlike terrestrial crops, which generally exhibit lower carbon capture efficiency per unit of biomass, microalgae can fix up to 1.83 kg of CO_2_ per kg of dry biomass, making them particularly attractive biological platforms for CCUS-oriented bioprocesses (Razzak et al. 2024); Mohapatra et al. 2022).

In photosynthetic systems, most reported surface-active compounds correspond to extracellular polysaccharides (EPS) and glycolipid-rich fractions with emulsifying activity, rather than canonical biosurfactants such as rhamnolipids or lipopeptides typically produced by heterotrophic or anaerobic bacteria. These EPS-based materials exhibit surface and interfacial activity and have been proposed as functional biosurfactant analogues in CO_2_-driven phototrophic platforms (de Morais et al., 2019; Han et al. 2013; Laroche 2022; Zhou et al. 2024).

Genetic strategies to improve carbon fixation and product formation include carboxysome engineering, overexpression of RuBisCO and carbonic anhydrase, and optimization of CCM-related genes to enhance intracellular CO_2_ availability. At the technological level, advances include high-density photobioreactors, improved light distribution systems, and CO₂-enriched cultivation platforms aimed at increasing biomass productivity and carbon capture efficiency (Rajkumar et al. 2022).

However, quantitative data on biosurfactant titers under fully CO_2_-driven phototrophic conditions remain scarce. Reported productivities of surface-active EPS in cyanobacteria typically range from tens to a few hundreds of mg·L⁻^1^, which remain substantially lower than those achieved in anaerobic or heterotrophic biosurfactant systems (Laroche 2022).

Despite their ecological diversity, photosynthetic systems face challenges related to scalability, resource demand, and sensitivity to controlled operational parameters, such as light distribution, temperature, and CO_2_ concentration, under industrial cultivation conditions (Occhipinti et al. 2024). Compared to anaerobic CCUS platforms, photosynthetic systems are constrained by light availability, lower volumetric productivity, and limited biosurfactant diversity. In contrast, anaerobic chemoautotrophs and facultative anaerobes operate independently of light, reach higher cell densities in conventional bioreactors, and have demonstrated biosurfactant titers in the g·L⁻^1^ range.

These limitations create a clear rationale for exploring anaerobic microbial CO_2_ fixation and Microbial-CCUS (Box 1), which may overcome phototrophic bottlenecks while simultaneously enabling the production of high-value biomolecules. However, the absence of harmonized quantitative metrics across studies, particularly regarding biosurfactant titers, productivities, and growth-associated parameters under CO_2_-driven phototrophic conditions, currently prevents a robust and quantitative comparison with anaerobic CCUS platforms. This challenge reflects a broader methodological gap in the literature rather than a lack of technological potential, underscoring the need for standardized reporting frameworks in future studies.

### 3.2 Non-photosynthetic CO_2_ fixation

Non-photosynthetic microorganisms provide a complementary approach to CO_2_ fixation that is often associated with lower operational energy demand and simplified cultivation requirements, as these systems avoid the restrictions related to light distribution, photolimitation, and reactor geometry that typically restrict volumetric productivity in phototrophic platforms. In this context, energy efficiency can be assessed through indicators such as energy demand per mass of CO_2_ captured or converted, energy consumption per unit of biosurfactant produced, and life-cycle energy intensity. However, these metrics remain rarely standardized across the literature, since most studies emphasize metabolic capability or product titers while detailed evaluations of agitation, gas transfer, and downstream energy inputs are reported inconsistently.

These organisms exploit chemical energy from substrates such as hydrogen, reduced sulfur compounds, or ammonia, which makes them particularly relevant in anaerobic and extreme environments. Their metabolic versatility underpins several biochemical pathways that channel CO_2_ into central metabolites and value-added products, offering significant potential for carbon capture technologies (Gong et al. 2018). The major pathways are summarized in Table 1., while their functional significance is outlined below.

#### Reductive tricarboxylic acid (rTCA) cycle

First described in *Chlorobium limicola*, this reverse TCA cycle incorporates CO_2_ into intermediates such as acetyl-CoA and pyruvate via ATP-citrate lyase and related enzymes. The rTCA cycle is pivotal in light-limited environments like hydrothermal vents, exemplifying metabolic adaptation to extreme conditions (Evans et al. 1966; Kajla et al. 2022).

#### Wood–Ljungdahl (WL) pathway

Found in acetogens and methanogens such as *Clostridium thermoaceticum*, this pathway reduces CO_2_ to formate and carbon monoxide, which then combine with a methyl group to form acetyl-CoA. Its minimal ATP requirement makes it highly energy-efficient and suitable for industrial CO_2_ conversion into acetate, methane, or other precursors (Ljungdahl 1986).

#### 3-Hydroxypropionate (3HP) bicycle

Discovered in *Chloroflexus aurantiacus*, this dual-cycle process incorporates CO_2_ into glyoxylate via intermediates such as malonyl-CoA and 3-hydroxypropionate, involving multiple enzymatic steps. It is particularly advantageous for microorganisms in nutrient-limited or extreme habitats (Kajla et al. 2022; Strauss and Fuchs 1993).

#### 3-Hydroxypropionate/4-hydroxybutyrate (3HP/4HB) pathway

Identified in autotrophic archaea such as *Metallosphaera sedula*, this pathway carboxylates acetyl-CoA to malonyl-CoA and regenerates acetyl-CoA through intermediates including 4-hydroxybutyrate. It is highly effective under anaerobic, thermophilic, and acidic conditions (Berg et al. 2007; Choi et al. 2022).

#### Dicarboxylate/4-hydroxybutyrate (Di/4HB) cycle

Observed in extremophiles like *Ignicoccus hospitalis*, this cycle combines CO_2_ with acetyl-CoA to form pyruvate and oxaloacetate, which are subsequently recycled into succinyl-CoA and acetyl-CoA. Its energy-efficient design supports robust carbon fixation under resource-limited conditions (Gong et al. 2018; Huber et al. 2008).

Compared to photosynthetic organisms, non-photosynthetic microorganisms maintain the capacity to thrive in light-limited or extreme habitats while remaining fully compatible with conventional bioreactor infrastructures. Importantly, integrating phototrophic and chemotrophic strategies may provide complementary advantages, enabling resilient Microbial-CCUS frameworks that couple carbon assimilation with the production of value-added metabolites and expand opportunities for industrial biomanufacturing.

## 4. Microbial surfactants

**Fig. 2:**
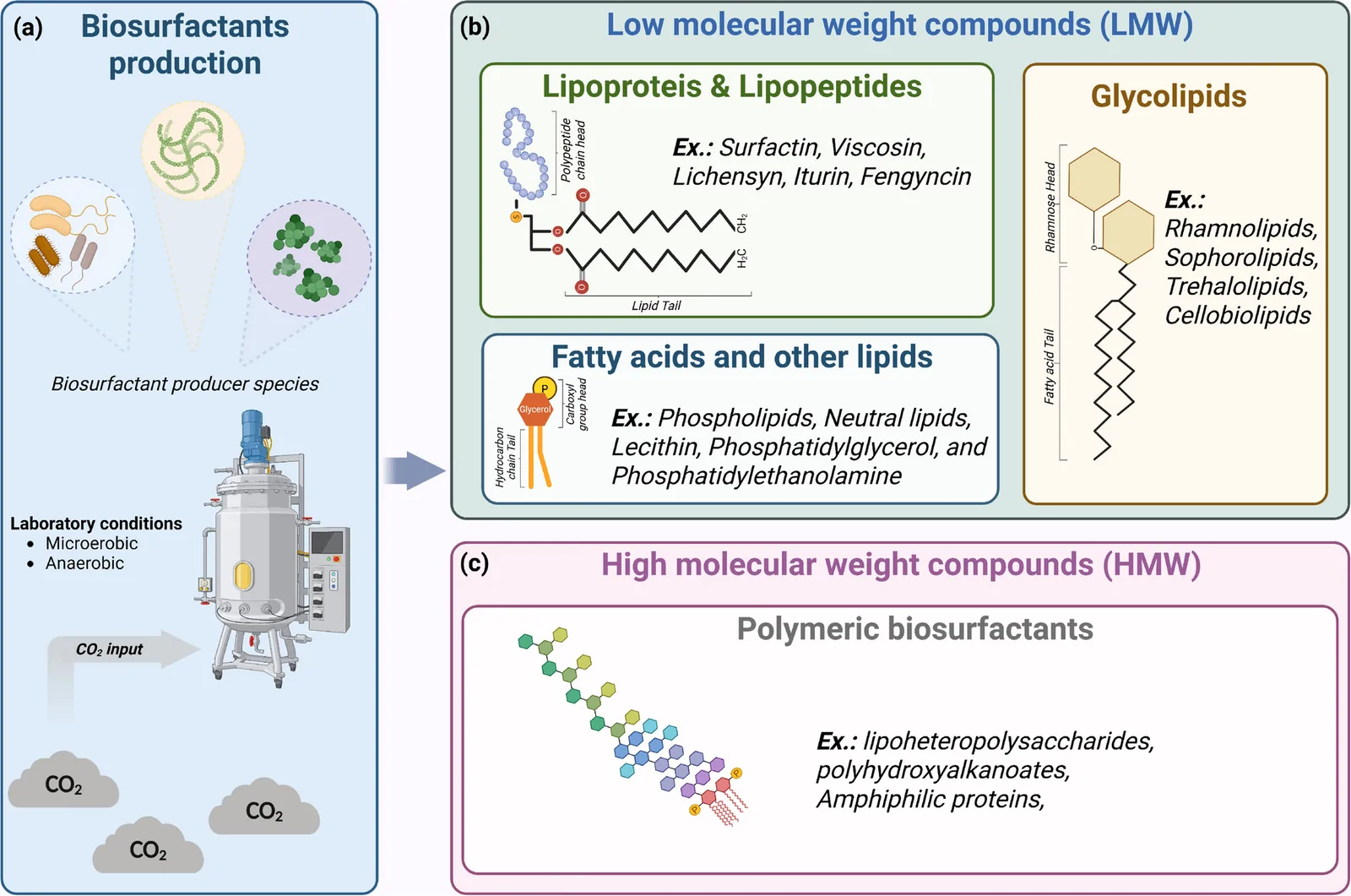
**Biosurfactants by classes**. Biosurfactants are naturally occurring compounds produced by microorganisms. **a)** Environmental and laboratory-cultured microbes serve as model systems for biosurfactant production under specific laboratory conditions, where CO_2_ can be utilized as a carbon source. CO_2_ can be utilized as a carbon source for generating biosurfactants. **b)** Microorganisms synthesize a variety of biosurfactant classes, typically composed of a hydrophilic “head” and a hydrophobic “tail,” with differences in their chemical composition. Based on their molecular weight and complexity, biosurfactants are categorized as low-molecular-weight (LMW) or **c)** high-molecular-weight (HMW)

Microorganisms capable of assimilating CO_2_ can channel fixed carbon into high-value biomolecules, offering a sustainable strategy for climate change mitigation. Among these products, biosurfactants stand out as amphiphilic metabolites capable of lowering surface and interfacial tension, solubilize hydrophobic substrates, and modulate cell–surface interactions. Their biodegradability, renewable origin, and eco-friendly properties make them attractive alternatives to synthetic surfactants (Kugaji et al. 2025; Markande et al. 2021). These molecules align with Microbial-CCUS concepts, where CO_2_ fixation is directly coupled to metabolite synthesis (Fig. 2a). Compared to synthetic surfactants, biosurfactants offer ecological advantages, including enhanced microbial motility, biofilm formation, competitive interactions, and resistance to toxic compounds (Gong et al. 2018; Hu et al. 2019).

### 4.1 Structural diversity and classification of biosurfactants

Biosurfactants are composed of a hydrophilic “head” (sugars, peptides, phosphates) and a hydrophobic “tail” (lipids or fatty acids). They are broadly classified into low-molecular-weight (LMW) and high-molecular-weight (HMW) compounds (Fig. 2b, c) (Qin et al. 2025). LMW biosurfactants (< 1500 Da) include glycolipids (e.g., rhamnolipids, sophorolipids), lipopeptides (surfactins, iturins, fengycins), fatty acids, and phospholipids. These molecules rapidly adsorb to interfaces, efficiently reducing water surface tension from 72 to ~ 27 mN·m^−1^ (Lakatos et al. 2022). Lipopeptides produced by *Bacillus* and *Pseudomonas* exhibit both surface-active and antimicrobial properties. HMW biosurfactants include polysaccharides, lipopolysaccharides, amphiphilic proteins, and polymeric complexes such as emulsan. These macromolecules act as potent emulsifiers, stabilizing biofilms and enhancing the bioavailability of hydrophobic substrates (Ibrahim et al. 2022).

The type and yield of biosurfactants obtained from bioprocesses are influenced by both the producing microorganism and the environmental conditions, including nutrient availability, oxygen levels, and salinity (Araújo et al., 2020; Moro et al. 2018; Napp et al. 2018). Understanding these parameters is essential for optimizing production and tailoring the physicochemical properties of biosurfactants to specific industrial applications.

### 4.2 Biosurfactant applications

**Fig. 3:**
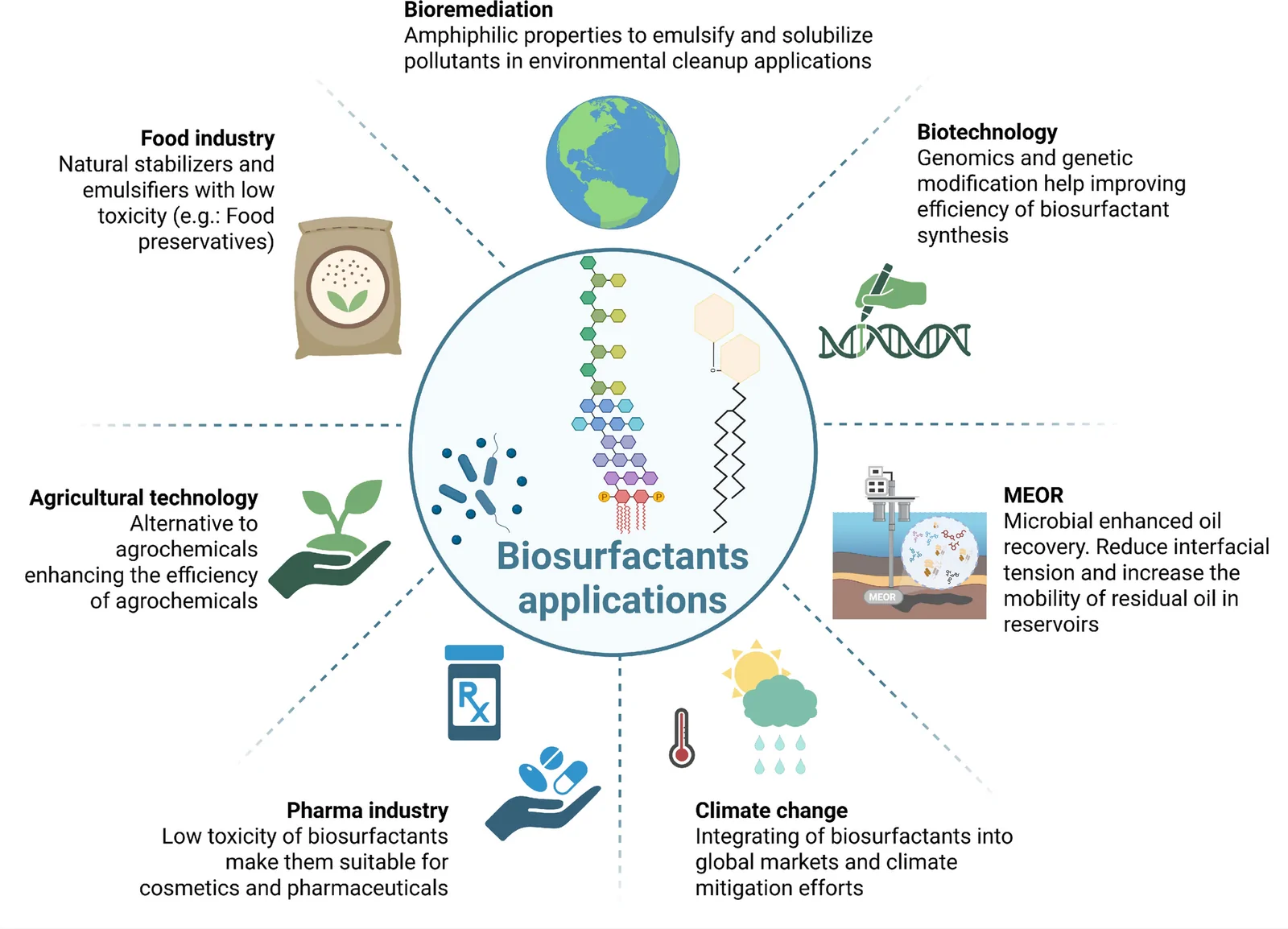
**Applications of biosurfactants.** Multisectoral applications of microbial biosurfactants and their relevance in environmental and industrial contexts. Due to their amphiphilic nature, biosurfactants play a key role in bioremediation, facilitating the emulsification and solubilization of hydrophobic pollutants. In agriculture, they serve as sustainable alternatives to agrochemicals, enhancing efficacy and reducing toxicity. In the food industry, biosurfactants are applied as natural preservatives and stabilizers. Their low toxicity and biodegradability enable their use in the pharmaceutical and cosmetic sectors. In energy systems, biosurfactants contribute to MEOR by improving oil mobilization. Advances in genomics and metabolic engineering further support biotechnological innovations to enhance biosurfactant yield and performance. Integration of biosurfactants into global markets may also support climate mitigation strategies by replacing synthetic surfactants with bio-based alternatives

The collective properties of biosurfactants make them sustainable alternatives to synthetic surfactants across multiple industries (Fig. 3). In environmental remediation, surfactins and glycolipids lower surface tension, promoting hydrocarbon solubilization and pollutant degradation, while polysaccharide-based compounds such as emulsan (*Acinetobacter calcoaceticus*) stabilize emulsions and increase the bioavailability of hydrophobic substrates, a key for oil recovery and bioremediation (Moro et al. 2018). In agriculture, lipopeptides such as iturins and fengycins act as antifungal agents and can trigger systemic resistance in plants, whereas phospholipids like lecithin and phosphatidylethanolamine serve as natural wetting and emulsifying agents, enhancing agrochemical efficiency while reducing synthetic inputs (Zhang et al. 2022). In the pharmaceutical and cosmetic sectors, surfactins support drug delivery and personal care formulations through strong surface activity and antimicrobial action, while phosphatidylglycerol enhances formulation stability under variable conditions, reinforcing the value of biosurfactants as safe multifunctional excipients (Sultan et al. 2025).

Despite this wide applicability, most biosurfactant production systems remain aerobic, and anaerobic pathways are largely unexplored. This knowledge gap reflects technical challenges related to the limited availability of oxygen-independent metabolic routes, low production yields, and constraints in energy and redox balance. Expanding biosurfactant synthesis to anaerobic or CO_2_-enriched systems could enhance process sustainability by reducing aeration costs, minimizing foaming, and enabling operation in closed bioreactors. Furthermore, coupling biosurfactant production with CO_2_ utilization offers the prospect of developing microbial platforms compatible with CCUS frameworks, where carbon capture and metabolite synthesis occur simultaneously, thereby broadening the scope of sustainable microbial biotechnology. The feasibility of anaerobic biosurfactant production has been demonstrated under specific environmental and physiological conditions.

### 4.3 Anaerobic biosurfactant production

The earliest evidence of biosurfactant synthesis under oxygen deprivation dates back to 1955, when *Desulfovibrio desulfuricans* was shown to produce surface-active compounds in the absence of oxygen (La Rivière 1955). In this pioneering study, cells were cultivated in a defined mineral medium containing sodium lactate as the sole carbon source, and anaerobiosis was achieved by boiling and cooling the medium before inoculation. Since this initial report, only a limited number of microbial taxa have been confirmed as capable of biosurfactant production under oxygen-limited or strictly anaerobic conditions (Eras-Muñoz et al. 2022; Hu et al. 2019).

Table 2 summarizes the bacterial isolates reported as anaerobic biosurfactant producers with demonstrated potential for CO_2_ fixation. Genera including *Bacillus*, *Pseudomonas*, and *Clostridium* stand out for their ability to reduce surface tension and interfacial tension and to produce structurally diverse biosurfactants under anoxic conditions. These organisms typically use organic acids, hydrocarbons, or CO_2_-derived intermediates as carbon sources, depending on their metabolic strategy. Table 2 highlights the substantial heterogeneity in experimental design and reporting across studies. Quantitative metrics such as productivity, growth rates, and biomass accumulation are inconsistently reported and often measured under non-comparable cultivation conditions. This lack of standardized performance indicators currently limits robust cross-study comparisons and represents a major bottleneck for evaluating the true potential of CO_2_-driven biosurfactant production within Microbial-CCUS frameworks.

**Table 2:** Overview of bacterial growth and biosurfactant production under oxygen-limited and anaerobic conditions

Microorganism	Biosurfactant type	Growth	Culture conditions	Carbon source	Growth-related metrics	Biosurfactant yield	Biosurfactant evidence	References
*D. desulfuricans* DSM 1926	Unknown	Anaerobic	Mineral medium 30 °C	Lactate	NR	NR	44–46 mN·m^−1^ ST Oil-spreading EOR 35%	(La Riviere et al., 1955)
*C. pasteurianum *NRRL B-598	Neutral lipid	Anaerobic	Modified Mallette medium Room temperature	Sucrose	NR	NR	30 mN·m^−1^ ST Foaming activity Relative CMC	(Cooper et al. 1980)
*B. mojavensis* JF-2	Lichenysin B	Anaerobic	Mineral medium 40 °C	Glucose	A_480_ 0.5–1.05	100–700 mg/L	27–31 mN·m^−1^ ST Foaming activity Relative CMC	(Javaheri et al. 1985)
*B. licheniformis* BAS50	Lichenysin A	Anaerobic	Mineral medium 40–45 °C	Glucose Sucrose	NR	160 mg/L	35 mN·m^−1^ ST CMC 12 mg/L	(Yakimov et al. 1995)
*B. licheniformis* BNP29; BNP36; Mep132	Lipopetide EPS	Anaerobic	CNY medium 50 °C	Crude oil	NR	50 mg/L 80–1600 mg/L	EOR 22%	(Yakimov et al. 1997)
*B. subtilis* ATCC 21332	Surfactin	Anaerobic	Mineral medium 32 °C	Glucose	Biomass 5.8–6 g/L	439 mg/L	HPLC	(Davis et al. 1999)
*B. licheniformis* *B. mojavensis*	Lipopetide	Anaerobic	Mineral medium 37 °C	Sucrose	NR	NR	Oil-spreading	(Youssef et al. 2005)
*Bacillus* sp*.* RS-1 *B. subtilis* subsp. *spizizenii* NRRL B-23049	Lipopetide	Anaerobic	Mineral medium 37 °C	Glucose	NR	90 mg/L	HPLC Relative ST Oil-spreading	(Youssef et al. 2007)
*B. licheniformis* VKM B-511	Lipopetide	Anaerobic	Mineral medium 30 °C	Kerosene	A_600_ 0.455	2590 mg/L	35.3 mN·m^−1^ ST E24 46.1%	(Gogotov and Miroshnikov 2009)
*B. mojavensis* GMTB-C1-2	Lipopetide	Anaerobic	Medium E 42 °C	Peptides Amino acids	A_600_ 0.650	NR	27 mN·m^−1^ ST E24 56–70%	(Ghojavand et al., 2011)
*B. subtilis* #191; #309; #311; #552; #573	Lipopetide Surfactin-like	Anaerobic	Mineral medium 40 °C	Hexadecane	Biomass 0.176–0.238 g/L	NR	30–41.5 mN·m^−1^ ST CMC 20–30 mg/L E24 13.3–34.2%	(Gudina et al., 2012)
*B. amyloliquefaciens* S499	Surfactins Iturins Fengycins	Oxygen-limited	RE medium 30° C	Glucose	A_600_ 0.9	252 mg/L; 108 mg/L; 10 mg/L	LC–ESI–MS	(Nihorimbere et al. 2012)
*Bacillus* sp. ISTS2	Lipopeptides Free fatty acids	Oxygen-limited	MSM medium 30 °C	CO_2_	NR	NR	GC–MS 33 mN·m^−1^ ST E24 53%	(Sundaram and Thakur 2015)
*Bacillus subtilis* DSM 10^ T^	Surfactin	Anaerobic	MSM medium 30 °C	Glucose	CDWmax 0.856 g/L	150 mg/L	HPLC	(Willenbacher et al. 2015)
*B. licheniformis* 421	Lichenysin G	Anaerobic	Synthetic seawater 50 °C	Molasses	A_600_ 1.2	9.60 mg/L	UHPLC-MS 0.60 mN·m^−1^ IFT	(Halim et al. 2017)
*Bacillus* sp. SS105	Lipopetide	Elevated CO_2_ conditions	MSM medium 30 °C	NaHCO_3_ Molasses	Biomass 2.78 g/L	2650 mg/L	FTIR and NMR Oil-spreading E24 63%	(Masheshwari et al., 2017)
*B. subtillis* ATCC 6633	Surfactin	Anaerobic	Mineral medium 37 °C	Glucose	NR	62 mg/L	FTIR IFT reduction	(Park et al. 2017)
*B. subtilis* JABs24; MG1; MG5	Surfactin	Anaerobic	Mineral medium 37 °C	Glucose	CDWmax 0.332 g/L–0.492 g/L	85.6–190 mg/L	HPLC	(Hoffmann et al. 2020)
*Bacillus subtilis* AnPL-1	Surfactin	Anaerobic	Mineral medium 39 °C	Sucrose	A_600_ 3.953	182.9 mg/L	28.5 mN·m^−1^ ST CMC 30 mg/L E24 70.5%	(Zhao et al. 2021a)
*B. licheniformis* EL3	Lipopetide	Oxygen-limited	MSM medium 37 °C	Glucose	Biomass 0.108 g/L	775 mg/g biomass	FTIR 29 mN·m^−1^ ST CMC 27 mg/L	(Leal et al. 2024)
*Pseudomonas* sp. BS2201 *Pseudomonas* sp. BS2203	Unknown	Anaerobic	PPGA medium 28 °C	Petroleum	NR	NR	38 mN·m^−1^ ST 34.7 mN·m^−1^ ST	(Grishchenkov et al. 2000)
*P. aeruginosa* PAO1T	Rhamnolipid	Anaerobic	Modified Davis medium 30 °C	Glycerol	NR	NR	TLC	(Nozawa et al. 2007)
*Pseudomonas* sp. ANBIOSURF-1	Glycolipid	Anaerobic	Mineral medium 30 °C	Coconut oil	NR	NR	FTIR and TLC CMC 52 mg/L	(Albino and Nambi 2010)
*P.aeruginosa* E03-40	Rhamnolipid	Oxygen-limited	Mineral medium 34 °C	Glycerol	NR	17 mg/g (dry cells)-h	Colorimetric analysis	(Pinzon et al. 2013)
*P. stutzeri* Rhl	Rhamnolipid	Anaerobic	Mineral medium 42 °C	Glycerol	NR	3120 mg/L	Colorimetric analysis EOR 15.7%	(Zhao et al. 2014)
*P. aeruginosa* SG	Rhamnolipid	Anaerobic	Mineral medium 37 °C	Glycerol	NR	1080 mg/L	FTIR and TLC 33.3 mN·m^−1^ ST CMC 80 mg/L E24 > 80%, EOR 8.33%	(Zhao et al. 2015a; 2015c; 2021b)
*P. aeruginosa* PrhlAB; PoprAB	Rhamnolipid	Anaerobic	Mineral medium 37 °C	Glycerol	NR	2420–3560 mg/L	37.2 mN·m^−1^ ST	(Zhao et al. 2015c)
*P. stutzeri* WJ-1	Rhamnolipid	Anaerobic	GN medium 37 °C	Glycerol	Biomass 5.1 g/L	390 mg/L	Colorimetric analysis 33 mN·m^−1^ ST	(Zhao et al. 2015b)
*P. stutzeri* CX3	Glycolipid Lipopeptides	Anaerobic	Mineral medium 30 °C	Glycerol	Biomass 2.6 g/L	NR	FTIR, TLC and GC-FID 30.4 mN·m^−1^ ST CMC 35 mg/L	(Fan et al. 2018)
*P. stutzeri* DQ3	Rhamnolipid	Anaerobic	Mineral medium 42 °C	Glycerol	NR	228 mg/L	33.8 mN·m^−1^ ST Oil-spreading E24 58%	(Zhao et al. 2018)
*P. putida* KT2440	Rhamnolipid	Anaerobic	Modified Wilms KPi medium 30 °C	Acetate	A_600_ 2–2.5	366–414 mg/L	Colorimetric analysis	(Widberger et al. 2024)
*P. aeruginosa* SGhm	Rhamnolipid	Anaerobic	GNP medium 35 °C	Glycerol	A_600_ 0.5–0.6	1540 mg/L	E24 89.4%	(Zhao et al. 2025)
*T. pseudethanolicus* 39E	Unknown	Anaerobic	Mineral medium 65 °C	Glucose Tryptone	A_660_ 0.8–1.2	NR	TLC	(Yen et al. 1991)
*Brevibacillus *sp. BS2202	Unknown	Anaerobic	PPGA medium 28 °C	Petroleum	NR	NR	31.6 mN·m^−1^ ST Oil-spreading	(Grishchenkov et al. 2000)
*A. thermohalophila* Fru22	Unknown	Anaerobic	Mineral medium 50 °C	Hexadecane Glucose	NR	NR	SDS-PAGE Relative ST reduction	(Denger et al. 2002)
*G. pallidus* H9	Glycolipopeptide	Anaerobic	Minimal medium 65 °C	Crude oil	Biomass 9.11 g/L	2160 mg/L	32.25 mN·m^−1^ ST CMC 22 mg/L E24 10–35%	(Wenjie et al., 2012)
*R. ruber* Z25	Unknown	Anaerobic	Mineral medium 37 °C	Paraffin	Biomass 0.11 g/L	530 mg/L	29.54 mN·m^−1^ ST 1.0 mN·m^−1^ IFT CMC 57–133 mg/L EOR 25.8%	(Zheng et al. 2012)
Fusant strain FA-2	Lipopeptide	Anaerobic	Medium E 37 °C	Sucrose	A_660_ 1.8	382 mg/L	TLC and LC–MS 31.2 mN·m^−1^ ST CMC 60 mg/L E24 50%, EOR 5.22%	(Liang et al. 2017)
*L. huabeiensis* HB-2	Lipopeptide	Anaerobic	Fermentation medium 40 °C	Glucose	A_660_ 0.830	300–400 mg/L	FTIR and TLC E24 40%, EOR 11%	(Ke et al. 2018)
*C. daeguensis* HB-4	Unknown	Anaerobic	Mineral medium 37 °C	Glucose	NR	NR	EOR 20.5%	(Ke et al. 2019)
*H. zhaodongensis* 253	Unknown	Oxygen-limited	Mineral medium 28 °C	CO_2_	NR	NR	E24 68.5%	(Santos et al. 2024)
*E. alkaliphilum* 283	Unknown	Oxygen-limited	Mineral medium 28 °C	CO_2_	NR	NR	E24 67.3%	(Santos et al. 2024)
*M. hydrocarbonocalsticus* and *Thalssopspira* sp. (consortia)	Lipopeptide	Anaerobic	Seawater 37 °C	Diesel	NR	4200 mg/L	66.9 mN·m^−1^ ST CMC 3300 mg/L	(Wahby et al. 2025)
*E. proteobaterium* and *Desulfovibrio* sp. (consortia)	Lipopeptide	Anaerobic	Seawater 37 °C	Diesel-sodium lactate	NR	4200 mg/L	66.5 mN·m^−1^ ST CMC 3400 mg/L E24 45.8%	(Wahby et al. 2025)
*Agarivorans* sp*.* and MVP-15 unclassified (consortia)	Lipopeptide	Anaerobic	Seawater 37 °C	Diesel-glucose	NR	5300 mg/L	47.3 mN·m⁻^1^ ST CMC 4200 mg/L E24 58.4%	(Wahby et al. 2025)
*Marinobacter* sp. and *Vibrio* sp. (consortia)	Lipopeptide	Anaerobic	Seawater 37 °C	Diesel	NR	4400 mg/L	33.4 mN·m^−1^ ST CMC 3500 mg/L	(Wahby et al. 2025)
*Desulfocurvus thunnarius* and *D. hontreensis* (consortia)	Lipopeptide	Anaerobic	Seawater 37 °C	Diesel-sodium lactate	NR	6400 mg/L	55.5 mN·m^−1^ ST CMC 3000 mg/L E24 46.8%	(Wahby et al. 2025)
*Agarivorans* sp. and *D. hontreensis *(consortia)	Lipoprotein	Anaerobic	Seawater 37 °C	Diesel-glucose	NR	6600 mg/L	49.8 mN·m^−1^ ST CMC 3000 mg/L E24 45.7%	(Wahby et al. 2025)

NR: not reported. Where quantitative metrics were unavailable. CDWmax: maximum cell dry weight, EOR: enhanced oil recovery, E24: emulsification index.

#### *Bacillus*

Among facultative anaerobes, *Bacillus* represents the most extensively documented genus for biosurfactant production under oxygen deprivation. Well-characterized isolates such as *B. mojavensis* JF-2 (formerly *B. licheniformis*) produce the lipopeptide lichenysin under strictly anaerobic conditions, achieving surface tension values below 30 mN·m^−1^ (Folmsbee et al. 2006; Javaheri et al. 1985). Subsequent studies on *B. licheniformis* strains (e.g., BAS50 and the BNP series) confirmed anaerobic lipopeptide synthesis with efficiencies approaching those of commercial surfactants (Yakimov et al. 1995, 1997). In some cases, anaerobic cultivation yielded higher biosurfactant titers than aerobic conditions (2.59 g/L vs. 0.69 g/L), although these comparisons remain study-specific and dependent on cultivation parameters (Gogotov & Miroshnikov 2009).

Oxygen-limited cultivation has also shown reduced excessive foaming, a critical bottleneck in industrial-scale-up biosurfactant production (Hoffmann et al. 2020). Recent evidence further indicates that oxygen limitation, rather than strict anaerobiosis, can represent an optimal metabolic window for *Bacillus*. Leal et al. (2024) demonstrated that *Bacillus* isolates maintain high lipopeptide productivity and achieve surface tension values below 30 mN·m^−1^ under oxygen-limited conditions, combining reduced foaming with enhanced process stability and extracellular biosurfactant accumulation.

Nevertheless, robust biosurfactant production under strictly anaerobic conditions remains uncommon. A large screening involving 207 bacterial strains revealed that only 35 tolerated anaerobiosis in the presence of 5% NaCl, with biosurfactant production under strictly anaerobic conditions restricted to *B. mojavensis* and *B. licheniformis* (Youssef et al. 2005). Subsequent field-scale evidence demonstrated that selected *Bacillus* strains not only tolerate but actively produce lipopeptide biosurfactants under reservoir anaerobic conditions. In a limestone petroleum reservoir, *Bacillus* strain RS-1 and *B. subtilis* subsp. *spizizenii* produced lipopeptides at average concentrations of 90 mg L^−1^, exceeding the critical micelle concentration (CMC) required for oil mobilization, with confirmed nitrate-respiring metabolism and carbon-balanced anaerobic fermentation (Youssef et al. 2007). These findings emphasize both the challenge of identifying robust anaerobic producers and the unique ecological and biotechnological value of *Bacillus* as an effective production chassis.

Mechanistic and genomic evidence further support a functional coupling between CO_2_ metabolism and biosurfactant synthesis in *Bacillus*. At the physiological level, anaerobic cultivation of *B. licheniformis* 421 revealed that despite low lichenysin G titers in the aqueous phase, pronounced reductions in interfacial tension and stable emulsion formation were achieved through bacterial accumulation at the oil–water interface, highlighting the importance of localized and cell-bound biosurfactant activity under oxygen deprivation (Halim et al. 2017). Consistent with this physiological behavior, enzymatic activities such as carbonic anhydrase and RuBisCO, together with biosynthetic markers (e.g., licA3, lipP1, C23O), suggest integration of carbon assimilation pathways and lipopeptide production (Phetcharat et al. 2019; Sundaram & Thakur 2015). Moreover, halotolerant and thermotolerant isolates, including oilfield-derived *B. subtilis* strains and *B. mojavensis* GMTB-C1-2, thrive under elevated salinity and temperature, while reference strains (ATCC 21332/6633) demonstrate anaerobic surfactin synthesis applied to interfacial control in CO_2_–brine systems (Davis et al. 1999; Ghojavand et al., 2011; Guez et al. 2008; Park et al. 2017). Process innovations reported in Table 2 include nitrate-respiring media (e.g., *B. subtilis* AnPL-1 producing around 150 mg/L surfactin on sucrose), foam-free bioreactor operation (*B. subtilis* JABs24), and oxygen-limited production of multiple lipopeptide families (*B. amyloliquefaciens* S499, producing surfactins, iturins, and fengycins) (Gudiña et al., 2012; Hoffmann et al. 2020; Maheshwari et al. 2017; Nihorimbere et al. 2012; Willenbacher et al. 2015; Zhao, et al. 2021a). Collectively, these findings establish *Bacillus* as a priority microbial chassis for anaerobic biosurfactant-CO_2_ coupling, with actionable optimization levers including oxygen and electron-acceptor management, CO_2_/bicarbonate supply, and fatty-acid composition.

#### *Pseudomonas*

Species of *Pseudomonas* combine metabolic versatility with the ability to synthesize glycolipid biosurfactants under anaerobic conditions, primarily through nitrate respiration (Zhao, et al. 2015a). Strains isolated from petroleum-contaminated environments (e.g., BS2201 and BS2203) reduce surface tension to 34–38 mN·m^−1^ under nitrate-reducing conditions (Grishchenkov et al. 2000), while *P. aeruginosa* produces rhamnolipids during hydrocarbon catabolism (Chayabutra et al. 2001). Anaerobic growth has also been reported under high-CO_2_ atmospheres, where biosurfactants contribute directly to cellular adaptation and membrane stabilization (Nozawa et al. 2007), with production demonstrated in strains such as ANBIOSURF-1 cultivated on vegetable oils (Albino & Nambi 2010). Similarly, the environmental isolate CX3 exhibits rhamnolipid production under anaerobic, nitrate-reducing conditions, further supporting the ecological relevance of glycolipid biosurfactant synthesis by naturally occurring *Pseudomonas* strains in oxygen-depleted environments (Fan et al. 2018).

Advances in bioprocess engineering have significantly improved anaerobic rhamnolipid production, including immobilized-cell systems for continuous operation (Pinzon et al. 2013), nitrate-respiring bioreactors operated at elevated temperatures (Zhao et al. 2018), and targeted genetic modification of biosynthetic pathways (Zhao et al. 2015a, c, 2021b). These approaches have achieved biosurfactant titers above 1.6 g/L under oxygen-limited conditions (Zhao et al. 2014). Moreover, co-culture systems sustain production in sulfide-rich oilfield environments (Zhao et al. 2015b), highlighting the resilience of *Pseudomonas* under geologically relevant stresses.

Notably, genetic studies in *Pseudomonas aeruginosa* indicate that efficient anaerobic rhamnolipid production is not an inherent physiological trait but rather the result of targeted metabolic engineering. In the SG strain, the wild-type background exhibits limited biosurfactant synthesis under nitrate-respiring conditions, whereas substantial yield improvements are achieved only after overexpression of key biosynthetic operons (rhlAB and rmlBDAC). These findings identify transcriptional regulation and precursor supply as primary bottlenecks for anaerobic secondary metabolism, while also demonstrating that enhanced biosurfactant titers can be attained without proportional increases in biomass, reinforcing the decoupling between growth and product formation under oxygen limitation (Zhao et al. 2015a, c; Zhao et al. 2025).

As summarized in Table 2, these attributes position *Pseudomonas* as a promising platform for integrating anaerobic biosurfactant production with CO_2_-rich industrial streams.

#### *Clostridium* and other related taxa

Beyond facultative anaerobes, several obligate anaerobic and extremophilic taxa further expand the phylogenetic and metabolic scope of anaerobic biosurfactant production. Early studies identified *Clostridium pasteurianum* and *Thermoanaerobacter pseudethanolicus* 39E as pioneer models secreting low-molecular-weight surface-active compounds under thermophilic, halotolerant, and strictly anaerobic conditions (Cooper et al. 1980; Yen et al. 1991). In contrast to fermentative anaerobes, taxa with distinct metabolic strategies have demonstrated even more pronounced interfacial performance. In this context, *Rhodococcus ruber* Z25 achieved exceptional interfacial performance (IFT 1.0 mN·m^−1^), enhanced oil recovery up to 25.8% during MEOR trials (Zheng et al. 2012). However, the thermophilic bacterium *Geobacillus pallidus* H9 exhibited a pronounced dependence on oxygen availability, with biosurfactant yields decreasing from 9.8 to 2.8 g/L under oxygen-limited conditions across temperatures of 45–80 °C and salinities up to 15% (Wenjie et al., 2012). Rather than representing an anaerobic advantage, this response highlights oxygen sensitivity as a critical constraint in certain thermophilic systems. Conversely, other extremophiles retain functional biosurfactant activity precisely under oxygen limitation. Complementarily, the alkaliphilic isolate *Exiguobacterium alkaliphilum* 283 exhibited pronounced emulsification activity under oxygen-limited conditions at elevated pH, underscoring the role of surface-active compounds in interfacial stabilization and stress adaptation in extreme environments (Santos et al. 2024).

Among halophilic systems, *Halomonas stevensii* efficiently coupled CO_2_ fixation with biosurfactant production, removing up to 98% of inlet CO_2_ under thiosulfate-driven metabolism and growing across broad salinity, pH, and temperature ranges (Mishra et al. 2017). Similarly, *Halomonas zhaodongensis* strain 253 sustained biosurfactant production under CO_2_-enriched (5–10%) and oxygen-limited conditions, maintaining emulsification indices around 60% and encoding carbonic anhydrase genes (γ-CA and CsoSCA) that facilitate CO_2_ hydration and bicarbonate utilization (Santos et al. 2024). Additional taxa, including *Brevibacillus* sp. BS2202, *Anaerophaga thermohalophila* Fru22, *Luteimonas huabeiensis* HB-2, and *Chelatococcus daeguensis* HB-4, further demonstrate biosurfactant-mediated interfacial modification and oil recovery under oxygen-deprived conditions (Denger et al. 2002; Grishchenkov et al. 2000; Ke et al. 2018, 2019).

Extending the single-strain perspective, Wahby et al. (2025) demonstrated that anaerobic biosurfactant functionality can also emerge robustly at the community level. Microbial consortia cultivated in diesel–sodium lactate and diesel–glucose systems produced biosurfactants with emulsification indices (E24) of 45.7–46.8%, CMC around 3.0 g/L, and relatively high biosurfactant titers (6.4–6.6 g·L⁻^1^), while achieving surface tension values of 49.8–55.5 mN·m⁻^1^ under oxygen-depleted conditions. Community composition analyses revealed mixed groups dominated by fermentative, hydrocarbon-degrading, and sulfate-reducing taxa, including *Marinobacter hydrocarbonoclasticus*, *Thalassospira* sp., *Epsilon proteobacteria*, *Desulfovibrio* sp., *Desulfocurvus thunnarius*, *Agarivorans* sp., and *Vibrio* sp., alongside several previously unreported biosurfactant-producing genera. Importantly, the functional interfacial performance of these consortia did not depend on exceptional surface tension reduction by individual members but rather on metabolic complementarity and cooperative interactions, highlighting community-level resilience and sustained emulsification capacity under reservoir-like anaerobic conditions.

Recent methodological advances, such as microplate-based anaerobic screening of oilfield samples, have accelerated the identification of new biosurfactant producers with surface tension values below 45 mN·m^−1^ (Zhao et al. 2022). At the process level, integrated systems coupling chemolithoautotrophic CO_2_ fixation (e.g., *Acetobacterium woodii*) with heterotrophic rhamnolipid synthesis by *Pseudomonas putida* illustrate emerging strategies for linking CO_2_ capture and biosurfactant production within unified platforms (Widberger et al. 2024). Overall, these taxa collectively demonstrate that anaerobic biosurfactant-CO_2_ coupling is a broadly distributed and mechanistically versatile trait, spanning facultative and obligate anaerobes as well as extremophilic lineages.

## 5. Challenges and future directions

Despite increasing industrial demand for sustainable surfactants, anaerobic biosurfactant production linked to CO_2_ fixation remains constrained by high costs, scale-up challenges, and downstream processing bottlenecks (Farias et al. 2021). In anoxic systems, slower microbial growth, inefficient substrate conversion, and yield variability further exacerbate these barriers. A major limitation lies in the scarcity of direct evidence for CO_2_/HCO_3_^−^ incorporation into biosurfactant precursors. Only a few isolates, such as *Halomonas* species, have shown promising emulsification under CO_2_-enriched conditions, though metabolic fluxes remain poorly resolved (Mishra et al. 2017; Santos et al. 2024). Addressing this knowledge gap requires not only improving biosurfactant yield but also mechanistically linking CO_2_ fixation pathways to surfactant biosynthesis within a Microbial-CCUS framework.

Recent studies have started to bridge this divide through genetic and process innovations. Hybrid or synthetic strains, such as the FA-2 protoplast fusion (*Bacillus mojavensis* × *Pseudomonas stutzeri*), have demonstrated lipopeptide production under anaerobiosis (382 mg/L), suggesting that engineered systems can channel inorganic carbon into amphiphilic molecules (Liang et al. 2017). Advances in high-throughput screening methods (e.g., microplate assays, oil displacement tests) now enable rapid strain discovery and preliminary tracking of carbon flux into glycolipid and lipopeptide backbones (Zhao et al. 2022; Widgerber et al., 2024).

At the technological level, bioreactor innovations are essential for ensuring efficient gas transfer, maintaining redox balance, and replicating subsurface pressures to sustain continuous CO_2_ uptake in biosurfactant-producing cultures. In addition, prolonged operation under high cell density and complex feed streams may promote biofilm formation and biofouling on reactor surfaces, potentially affecting mass transfer efficiency and maintenance requirements. Although not intrinsic to biosurfactant metabolism, such phenomena represent practical engineering limitations that must be addressed to ensure stable long-term Microbial-CCUS performance (Hoffmann et al. 2020; Zhao et al. 2018).

Functional characterization of CO_2_-derived biosurfactants, evaluating their CMC, stability, toxicity, biodegradability, and performance under saline or high-pressure conditions will ultimately determine their competitiveness with synthetic analogs and conventional aerobic products (Zhang et al. 2022).

Furthermore, computational tools and AI-driven metabolic design are redefining microbial carbon utilization optimization. Genetic algorithm-based AI and sequence-based predictors, such as Evo and ProGen, can analyze vast genomic datasets to reveal functional gene networks and optimize pathways for biosurfactant biosynthesis and CO_2_ fixation (Januszewski & Jain 2024; Nguyen et al. 2024; Sasse et al. 2024). Integrating AI-guided predictions with microbial genetics expertise offers a powerful route for targeted pathway design, reduced experimental effort, and accelerated strain optimization (Goodswen et al. 2021; Simon et al. 2024).

Ultimately, techno-economic and life-cycle assessments must determine whether CO_2_-derived biosurfactants can compete not only with synthetic surfactants but also with conventional microbial ones (Sarubbo et al. 2022). By coupling the discovery of novel isolates with verified CO_2_ incorporation, advanced genetic engineering, and tailored cultivation systems, this emerging field can expand our understanding of microbial adaptation to anoxic niches while creating innovative Microbial-CCUS strategies that deliver both economic and environmental value. Collectively, these integrative efforts position CO_2_-derived biosurfactants as pivotal components of a low-carbon bioeconomy, embedding them within global climate mitigation and sustainable industrial frameworks (Eras-Muñoz et al. 2022; Kugaji et al. 2025; Sarubbo et al. 2022). Achieving this integration at scale ultimately depends on advances at the molecular level.

### 5.1 Genetic and synthetic biology approaches to integrate CO_2_ fixation and biosurfactant production

At the molecular level, recent progress in genetic and synthetic biology provides the foundation for directly coupling CO_2_ assimilation with biosurfactant biosynthesis. Although experimental demonstrations remain limited, the combination of pathway engineering, modular design, and genome editing is enabling microbial platforms that coordinate carbon fixation with surfactant production within the Microbial-CCUS paradigm (Coimbra et al. 2025; Lea-Smith et al. 2025).

Synthetic biology now allows the construction of artificial operons linking CO_2_-fixing modules to reconfigured metabolic routes, channeling assimilated carbon toward the synthesis of multicarbon compounds. For example, engineered *Escherichia coli* strains expressing the RuBisCO–PRK cycle have shown enhanced CO_2_ assimilation and increased flux toward succinate and amino acid precursors (Zhou et al. 2025). Similarly, modular systems with optimized promoters, ribosome-binding sites, and artificial carboxysomes enable spatial coordination between carbon fixation and downstream biosynthesis. The inclusion of RuBisCO activator proteins in recombinant carboxysomes significantly improves enzyme activation and CO_2_ capture efficiency, extending the applicability of metabolic engineering for tailored compound production (Chen et al. 2022; Liu et al. 2022).

In addition to autotrophic models, the application of CRISPR-Cas systems to organisms such as *Cupriavidus necator* and anaerobic acetogens like *Clostridium autoethanogenum* has expanded the potential for carbon fixation through genetically tractable bacterial chassis. In particular, facultative or obligate anaerobes represent ideal platforms for anaerobic Microbial-CCUS. These genome-scale interventions allow for precise redirection of metabolic fluxes toward amphiphilic and lipidic products, including biosurfactant precursors (Yu et al. 2022). Extending these strategies to anoxic or microaerophilic bacteria remains a promising yet underexplored direction for true CO_2_-driven biosurfactant production.

Complementary computational frameworks are accelerating this progress. Machine learning platforms such as the Automated Recommendation Tool (ART) assist in predicting optimal genetic modifications and pathway rewiring for targeted compound synthesis (Chen & Xia 2023). Furthermore, recent reviews emphasize that robust microbial cell factories designed for sustainable manufacturing depend on finely tuned metabolic engineering to enhance the synthesis of multicarbon and lipophilic metabolites (Yan et al. 2024). Together, these genetic, synthetic, and computational strategies delineate a realistic roadmap toward next-generation microbial cell factories capable of converting inorganic carbon into high-value amphiphilic molecules, aligning biomanufacturing innovation with climate change mitigation and circular carbon economy goals.

## 6. Conclusions

The exploration of biosurfactants derived from microbial CO_2_ capture represents a promising opportunity to address urgent environmental challenges while advancing sustainable industrial practices. These compounds offer eco-friendly and biodegradable alternatives to synthetic surfactants, with potential applications in environmental remediation, agriculture, pharmaceuticals, and cosmetics. Both photosynthetic and non-photosynthetic bacteria provide suitable model organisms for microbial CCUS strategies. Advances in genetic engineering and systems biology enable the optimization of biosynthetic pathways, improved yields, and greater economic feasibility. Furthermore, integrating artificial intelligence into biosurfactant research holds additional potential to enhance process efficiency and resource utilization.

To date, quantitative data comparing CO_2_ fixation rates and biosurfactant yields under integrated microbial-CCUS systems remain scarce, as most studies focus either on CO_2_ assimilation or on biosurfactant productivity in isolation. Despite these advances, microbial CO_2_ capture and biosurfactant synthesis technologies remain at an early stage of development, typically between Technological Readiness Levels (TRL) 3 and 4. Most studies have been conducted under laboratory or small pilot conditions, where process efficiency, energy balance, and product recovery costs still represent major uncertainties. Achieving economic viability will require improving CO_2_ fixation rates and biosurfactant yields through metabolic engineering, as well as ensuring access to low-cost CO_2_ sources and renewable feedstocks. Integrating microbial CCUS processes with existing industrial emitters offers a promising route to reduce operational costs and accelerate scale-up. In parallel, comprehensive life cycle and techno-economic assessments will be essential to benchmark these systems against conventional petrochemical surfactants. Progressing toward TRL 6–7 will demand demonstration-scale facilities capable of continuous operation, validation of technical performance, and the implementation of policy instruments—such as carbon credits and green certification schemes—to foster market acceptance.

Future integration of CO_2_ assimilation with industrial infrastructures, alongside process optimization, is expected to facilitate progressive scale-up and greater economic feasibility of microbial CCUS platforms. However, widespread deployment remains constrained by challenges related to scalability, process optimization, and cost competitiveness. Overcoming these limitations will require coordinated efforts among academia, industry, and policymakers, supported by targeted incentives, regulatory frameworks, and multidisciplinary collaboration. Achieving these advancements could ultimately enable the deployment of sustainable microbial CCUS platforms, establish economically viable biosurfactant production at scale, drive greener industrial practices, contribute to global carbon reduction, and promote a circular bioeconomy that aligns environmental stewardship with economic growth.

## Data Availability

Not applicable.

## References

[CR1] Al-Ghussain L (2019) Global warming: review on driving forces and mitigation. Environ Prog Sustain Energy 38:13–21. 10.1002/ep.13041

[CR2] Albino JD, Nambi IM (2010) Partial characterization of biosurfactant produced under anaerobic conditions by *Pseudomonas* sp. ANBIOSURF-1. Adv Mater Res 93:623–626. 10.4028/www.scientific.net/AMR.93-94.623

[CR3] Ángeles R, Arnaiz E, Gutiérrez J, Muñoz R, Lebrero R (2021) Biogas-based production of glycogen by *Nostoc muscorum*: assessing the potential of transforming CO_2_ into value-added products. Chemosphere 275:129885. 10.1016/j.chemosphere.2021.129885 33636520 10.1016/j.chemosphere.2021.129885

[CR4] Angermayr SA, Hellingwerf KJ, Lindblad P, de Teixeira Mattos MJ (2009) Energy biotechnology with cyanobacteria. Curr Opin Biotechnol 20(3):257–263. 10.1016/j.copbio.2009.05.011 19540103 10.1016/j.copbio.2009.05.011

[CR5] Araújo WJ, Oliveira JS, Araújo SCS, Minnicelli CF, Silva-Portela RCB, da Fonseca MMB, Freitas JF, Silva-Barbalho KK, Napp AP, Pereira JES, Peralba MCR, Passaglia LMP, Vainstein MH, Agnez-Lima LF (2020) Microbial Culture in Minimal Medium With Oil Favors Enrichment of Biosurfactant Producing Genes. Front Bioeng Biotechnol Vol 8-2020. https://www.frontiersin.org/journals/bioengineering-and-biotechnology/articles/10.3389/fbioe.2020.00962 10.3389/fbioe.2020.00962 PMC7431673 32850771

[CR6] Ataeian M, Liu Y, Canon-Rubio KA, Nightingale M, Strous M, Vadlamani A (2019) Direct capture and conversion of CO_2_ from air by growing a cyanobacterial consortium at pH up to 11.2. Biotechnol Bioeng 116:1604–1611. 10.1002/bit.26974 30906982 10.1002/bit.26974 PMC6593468

[CR7] Bardi MJ, Müller F, Polag D, Gabbiye Habtu N, Koch K (2025) The intriguing effect of CO_2_ enrichment in anaerobic digestion. Bioresour Technol 416. 10.1016/j.biortech.2024.131743 10.1016/j.biortech.2024.131743 39500399

[CR8] Bassham JA, Benson AA, Kay LD, Harris AZ, Wilson AT, Calvin M (1954) The path of carbon in photosynthesis. XXI. The cyclic regeneration of carbon dioxide acceptor. J Am Chem Soc 76:1760–1770. 10.1021/ja01636a012

[CR9] Berg IA, Kockelkorn D, Buckel W, Fuchs G (2007) A 3-hydroxypropionate/4-hydroxybutyrate autotrophic carbon dioxide assimilation pathway in Archaea. Science 318:1782–1786. 10.1126/science.1149976 18079405 10.1126/science.1149976

[CR10] Brazzola N, Meskaldji A, Patt A, Tröndle T, Moretti C (2025) The role of direct air capture in achieving climate-neutral aviation. Nat Commun 16:—. 10.1038/s41467-024-55482-6 39799106 10.1038/s41467-024-55482-6 PMC11724844

[CR11] Calvin K, Dasgupta D, Krinner G, Mukherji A, Thorne PW, Trisos C, Romero J, Aldunce P, Barrett K, Blanco G, Cheung WWL, Connors S, Denton F, Diongue-Niang A, Dodman D, Garschagen M, Geden O, Hayward B, Jones C, Ha M (2023) Climate change 2023: synthesis report. Contribution of WGs I, II and III to the Sixth Assessment Report of the IPCC. IPCC, Geneva. 10.59327/IPCC/AR6-9789291691647

[CR12] Chayabutra C, Wu J, Ju L-K (2001) Rhamnolipid production by *Pseudomonas aeruginosa* under denitrification: effects of limiting nutrients and carbon substrates. Biotechnol Bioeng 72:25–33. 10.1002/1097-0290(20010105)72:1<25::AID-BIT4>3.0.CO;2-J 11084590 10.1002/1097-0290(20010105)72:1<25::aid-bit4>3.0.co;2-j

[CR13] Chen PR, Xia PF (2023) Carbon recycling with synthetic CO_2_ fixation pathways. Curr Opin Biotechnol 85:103023. 10.1016/j.copbio.2023.103023 38007984 10.1016/j.copbio.2023.103023

[CR14] Chen T, Fang Y, Jiang Q, Dykes GF, Lin Y, Price GD, Long BM, Liu L-N (2022) Incorporation of functional Rubisco activases into engineered carboxysomes to enhance carbon fixation. ACS Synth Biol 11:154–161. 10.1021/acssynbio.1c00311 34664944 10.1021/acssynbio.1c00311 PMC8787814

[CR15] Choi KR, Ahn Y-J, Lee SY (2022) Bacterial conversion of CO_2_ to organic compounds. J CO2 Util 58:101929. 10.1016/j.jcou.2022.101929

[CR16] Cobo S, Galán-Martín Á, Tulus V, Huijbregts MAJ, Guillén-Gosálbez G (2022) Human and planetary health implications of negative emissions technologies. Nat Commun 13:—. 10.1038/s41467-022-30136-7 35534480 10.1038/s41467-022-30136-7 PMC9085842

[CR17] Coimbra AAB, Prakash S, Jiménez JI, Rios-Solis L (2025) Establishing *Halomonas* as a chassis for industrial biotechnology: advances in synthetic biology tool development and metabolic engineering strategies. Microb Cell Fact 24:133. 10.1186/s12934-025-02757-2 40506695 10.1186/s12934-025-02757-2 PMC12164125

[CR18] Cooper DG, Zajic JE, Gerson DF (1980) Isolation and identification of biosurfactants produced during anaerobic growth of *Clostridium pasteurianum*. J Ferment Technol 58:83–86

[CR19] Correa SS, Schultz J, Zahodnik-Huntington B, Naschberger A, Rosado AS (2025) Carboxysomes: the next frontier in biotechnology and sustainable solutions. Biotechnol Adv 79:108511. 10.1016/j.biotechadv.2024.108511 39732444 10.1016/j.biotechadv.2024.108511

[CR20] Cuéllar-Franca RM, Azapagic A (2015) Carbon capture, storage and utilisation technologies: a critical analysis and comparison of their life cycle environmental impacts. J CO2 Util 9:82–102. 10.1016/j.jcou.2014.12.001

[CR21] Davis DA, Lynch HC, Varley J (1999) The production of surfactin in batch culture by *Bacillus subtilis* ATCC 21332 is strongly influenced by the conditions of nitrogen metabolism. Enzyme Microb Technol 25:322–329. 10.1016/S0141-0229(99)00048-4

[CR22] de Morais MG, de Morais EG, Duarte JH, Deamici KM, Mitchell BG, Costa JAV (2019) Biological CO2 mitigation by microalgae: technological trends, future prospects and challenges. World J Microbiol Biotechnol 35(5):78. 10.1007/s11274-019-2650-9 10.1007/s11274-019-2650-9 31087167

[CR23] de Oliveira Maciel A, Christakopoulos P, Rova U, Antonopoulou I (2022) Carbonic anhydrase to boost CO_2_ sequestration: improving CCUS. Chemosphere 299:134419. 10.1016/j.chemosphere.2022.134419 35364080 10.1016/j.chemosphere.2022.134419

[CR24] Della Valle S, Tu W, Huang WE (2024) Construction of microbial platform chassis for CO2 utilisation. Curr Opin Syst Biol 37:100489. 10.1016/j.coisb.2023.100489

[CR25] Denger K, Warthmann R, Ludwig W, Schink B (2002) *Anaerophaga thermohalophila* gen. nov., sp. nov., a moderately thermohalophilic, strictly anaerobic fermentative bacterium. Int J Syst Evol Microbiol 52:173–178. 10.1099/00207713-52-1-173 11837300 10.1099/00207713-52-1-173

[CR26] Duarte JH, de Morais EG, Radmann EM, Costa JAV (2017) Biological CO_2_ mitigation from coal power plant by *Chlorella fusca* and *Spirulina* sp. Bioresour Technol 234:472–475. 10.1016/j.biortech.2017.03.066 28342576 10.1016/j.biortech.2017.03.066

[CR27] Eras-Muñoz E, Farré A, Sánchez A, Font X, Gea T (2022) Microbial biosurfactants: a review of recent environmental applications. Bioengineered 13:12365–12391. 10.1080/21655979.2022.2074621 35674010 10.1080/21655979.2022.2074621 PMC9275870

[CR28] Evans MC, Buchanan BB, Arnon DI (1966) A new ferredoxin-dependent carbon reduction cycle in a photosynthetic bacterium. Proc Natl Acad Sci U S A 55:928–934. 10.1073/pnas.55.4.928 5219700 10.1073/pnas.55.4.928 PMC224252

[CR29] Fan F, Zhang B, Morrill PL, Husain T (2018) Isolation of nitrate-reducing bacteria from an offshore reservoir and the associated biosurfactant production. RSC Adv 8(47):26596–26609. 10.1039/C8RA03377C 35541051 10.1039/c8ra03377c PMC9083026

[CR30] Farias CBB, Almeida FCG, Silva IA, Souza TC, Meira HM, Soares da Silva RCF, Luna JM, Santos VA, Converti A, Banat IM, Sarubbo LA (2021) Production of green surfactants: market prospects. Electron J Biotechnol 51:28–39. 10.1016/j.ejbt.2021.02.002

[CR31] Folmsbee M, Duncan K, Han SO, Nagle D, Jennings E, McInerney M (2006) Re-identification of the halotolerant, biosurfactant-producing *Bacillus licheniformis* strain JF-2 as *Bacillus mojavensis* strain JF-2. Syst Appl Microbiol 29:645–649. 10.1016/j.syapm.2006.01.010 16488097 10.1016/j.syapm.2006.01.010

[CR32] Ghojavand H, Vahabzadeh F, Azizmohseni F (2011) A halotolerant, thermotolerant, and facultative biosurfactant producer: Identification and molecular characterization of a bacterium and evolution of emulsifier stability of a lipopeptide biosurfactant. Biotechnology and Bioprocess Engineering (BBE) 16(1):72–80. 10.1007/s12257-010-0148-2

[CR33] Gogotov IN, Miroshnikov AI (2009) Influence of growth medium composition and physicochemical factors on biosurfactant production by *Bacillus licheniformis* VKM B-511. Appl Biochem Microbiol 45:588–592. 10.1134/S0003683809060027 20067148

[CR34] Gong F, Zhu H, Zhang Y, Li Y (2018) Biological carbon fixation: from natural to synthetic. J CO2 Util 28:221–227. 10.1016/j.jcou.2018.09.014

[CR35] Goodswen SJ, Barratt JLN, Kennedy PJ, Kaufer A, Calarco L, Ellis JT (2021) Machine learning and applications in microbiology. FEMS Microbiol Rev 45:—. 10.1093/femsre/fuab015 33724378 10.1093/femsre/fuab015 PMC8498514

[CR36] Grishchenkov VG, Townsend RT, McDonald TJ, Autenrieth RL, Bonner JS, Boronin AM (2000) Degradation of petroleum hydrocarbons by facultative anaerobic bacteria under aerobic and anaerobic conditions. Process Biochem 35:889–896. 10.1016/S0032-9592(99)00145-4

[CR37] Gudiña EJ, Pereira JFB, Rodrigues LR, Coutinho JAP, Teixeira JA (2012) Isolation and study of microorganisms from oil samples for application in Microbial Enhanced Oil Recovery. Int Biodeterior Biodegrad 68:56–64. 10.1016/j.ibiod.2012.01.001

[CR38] Guez JS, Müller CH, Danze PM, Büchs J, Jacques P (2008) Respiration activity monitoring system (RAMOS) to study influence of oxygen transfer rate on lipopeptide synthesis by *Bacillus subtilis* ATCC6633. J Biotechnol 134:121–126. 10.1016/j.jbiotec.2008.01.003 18282625 10.1016/j.jbiotec.2008.01.003

[CR39] Halim AY, Nielsen SM, Nielsen KF, Lantz AE (2017) CPC testing: towards the understanding of microbial metabolism in relation to microbial enhanced oil recovery. J Pet Sci Eng 149:151–160. 10.1016/j.petrol.2016.10.031

[CR40] Han P-P, Sun Y, Wu X-Y, Yuan Y-J, Dai Y-J, Jia S-R (2013) Emulsifying, flocculating, and physicochemical properties of exopolysaccharide produced by cyanobacterium *Nostoc flagelliforme*. Appl Biochem Biotechnol. 10.1007/s12010-013-0505-7 24043454 10.1007/s12010-013-0505-7

[CR41] Hanifa M, Agarwal R, Sharma U, Thapliyal PC, Singh LP (2023) A review on CO2 capture and sequestration in the construction industry: emerging approaches and commercialized technologies. J CO2 Util 67:102292. 10.1016/j.jcou.2022.102292

[CR42] Hoffmann M, Fernandez Cano Luna DS, Xiao S, Stegemüller L, Rief K, Heravi KM, Lilge L, Henkel M, Hausmann R (2020) Towards the anaerobic production of surfactin using *Bacillus subtilis*. Front Bioeng Biotechnol 8:554903. 10.3389/fbioe.2020.554903 33324620 10.3389/fbioe.2020.554903 PMC7726195

[CR43] Hu G, Li Y, Ye C, Liu L, Chen X (2019) Engineering microorganisms for enhanced CO_2_ sequestration. Trends Biotechnol 37:532–547. 10.1016/j.tibtech.2018.10.008 30447878 10.1016/j.tibtech.2018.10.008

[CR44] Huber H, Gallenberger M, Jahn U, Eylert E, Berg IA, Kockelkorn D, Eisenreich W, Fuchs G (2008) A dicarboxylate/4-hydroxybutyrate autotrophic carbon assimilation cycle in the hyperthermophilic archaeum *Ignicoccus hospitalis*. Proc Natl Acad Sci U S A 105:7851–7856. 10.1073/pnas.0801043105 18511565 10.1073/pnas.0801043105 PMC2409403

[CR45] Ibrahim HAH, Abou Elhassayeb HE, El-Sayed WMM (2022) Potential functions and applications of diverse microbial exopolysaccharides in marine environments. J Genet Eng Biotechnol 20:151. 10.1186/s43141-022-00432-2 36318392 10.1186/s43141-022-00432-2 PMC9626724

[CR46] Ighalo JO, Dulta K, Kurniawan SB, Omoarukhe FO, Ewuzie U, Eshiemogie SO, Ojo AU, Abdullah SRS (2022) Progress in microalgae application for CO2 sequestration. Clean Chem Eng 3:100044. 10.1016/j.clce.2022.100044

[CR47] International Energy Agency (2021) Technology perspectives—special report on carbon capture, utilisation and storage: CCUS in clean energy transitions. www.iea.org/t&c/. Accessed 10 Nov 2025

[CR48] Januszewski M, Jain V (2024) Next-generation AI for connectomics. Nat Methods 21:1398–1399. 10.1038/s41592-024-02336-0 39122949 10.1038/s41592-024-02336-0

[CR49] Javaheri M, Jenneman GE, McInerney MJ, Knapp RM (1985) Anaerobic production of a biosurfactant by *Bacillus licheniformis* JF-2. Appl Environ Microbiol 50:698–700. 10.1128/aem.50.3.698-700.1985 16346889 10.1128/aem.50.3.698-700.1985 PMC238693

[CR50] Kajla S, Kumari R, Nagi GK (2022) Microbial CO_2_ fixation and biotechnology in reducing industrial CO_2_ emissions. Arch Microbiol 204:—. 10.1007/s00203-021-02677-w 35061105 10.1007/s00203-021-02677-w

[CR51] Kammerer S, Borho I, Jung J, Schmidt MS (2023) CO_2_ capturing methods of the last two decades. Int J Environ Sci Technol 20:8087–8104. 10.1007/s13762-022-04680-0

[CR52] Karimi M, Shirzad M, Silva JAC, Rodrigues AE (2023) Carbon dioxide separation and capture by adsorption: a review. Environ Chem Lett 21:2041–2084. 10.1007/s10311-023-01589-z 10.1007/s10311-023-01589-z PMC10018639 37362013

[CR53] Karishma S, Kamalesh R, Saravanan A, Deivayanai VC, Yaashikaa PR, Vickram AS (2024) Recent advancements in biochemical fixation and transformation of CO2 into constructive products: a review. Biochem Eng J 208:109366. 10.1016/j.bej.2024.109366

[CR54] Ke C-Y, Lu G-M, Wei Y-L, Sun W-J, Hui J-F, Zheng X-Y, Zhang Q-Z, Zhang X-L (2019) Biodegradation of crude oil by *Chelatococcus daeguensis* HB-4 and its potential for MEOR in heavy oil reservoirs. Bioresour Technol 287:121442. 10.1016/j.biortech.2019.121442 31085429 10.1016/j.biortech.2019.121442

[CR55] Ke C-Y, Sun W-J, Li Y-B, Lu G-M, Zhang Q-Z, Zhang X-L (2018) Microbial enhanced oil recovery in Baolige Oilfield using an indigenous facultative anaerobic strain *Luteimonas huabeiensis* sp. nov. J Pet Sci Eng 167:160–167. 10.1016/j.petrol.2018.04.015

[CR56] Khandelwal A, Anand A, Raghuvanshi S, Gupta S (2021) Integrated approach for microbial carbon dioxide (CO_2_) fixation process and wastewater treatment for the production of hydrocarbons: experimental studies. J Environ Chem Eng 9:105116. 10.1016/j.jece.2021.105116

[CR57] Kugaji M, Ray SK, Parvatikar P, Raghu AV (2025) Biosurfactants: strategies for economical production, applications and recent advancements. Adv Colloid Interface Sci 337:103389. 10.1016/j.cis.2024.103389 39765093 10.1016/j.cis.2024.103389

[CR58] Kumar M, Sundaram S, Gnansounou E, Larroche C, Thakur IS (2018) Carbon dioxide capture, storage and production of biofuel and biomaterials by bacteria: A review. In Bioresour Technol 247:1059–1068. Elsevier Ltd. 10.1016/j.biortech.2017.09.050 10.1016/j.biortech.2017.09.050 28951132

[CR59] La Rivière JWM (1955) The production of surface-active compounds by micro-organisms and its possible significance in oil recovery. Antonie Van Leeuwenhoek 21:9–27. 10.1007/BF02543795 14350597 10.1007/BF02543795

[CR60] Lakatos ES, Cioca LI, Szilagyi A, Vladu MG, Stoica RM, Moscovici M (2022) A systematic review on biosurfactants’ contribution to the transition to a circular economy. Processes 10.10.3390/pr10122647

[CR61] Laroche C (2022) Exopolysaccharides from microalgae and cyanobacteria: diversity of strains, production strategies, and applications. Mar Drugs. 10.3390/md20050336 35621987 10.3390/md20050336 PMC9148076

[CR62] Lea-Smith DJ, Hassard F, Coulon F, Partridge N, Horsfall L, Parker KDJ, Smith RDJ, McCarthy RR, McKew B, Gutierrez T, Kumar V, Dotro G, Yang Z, Curtis TP, Golyshin P, Heaven S, Jefferson B, Jeffrey P, Jones DL (2025) Engineering biology applications for environmental solutions: potential and challenges. Nat Commun 16:3538. 10.1038/s41467-025-58492-0 40229265 10.1038/s41467-025-58492-0 PMC11997111

[CR63] Leal E, Teixeira JA, Gudiña EJ (2024) Development of foam-free biosurfactant production processes using *Bacillus licheniformis*. Fermentation. 10.3390/fermentation10070340

[CR64] Li A, Cao X, Fu R, Guo S, Fei Q (2024) Biocatalysis of CO_2_ and CH_4_: key enzymes and challenges. Biotechnol Adv 72:108347. 10.1016/j.biotechadv.2024.108347 38527656 10.1016/j.biotechadv.2024.108347

[CR65] Liang X, Shi R, Radosevich M, Zhao F, Zhang Y, Han S, Zhang Y (2017) Anaerobic lipopeptide biosurfactant production by an engineered bacterial strain for in situ MEOR. RSC Adv 7:20667–20676. 10.1039/C7RA02453C

[CR66] Liu X, Li L, Zhao G, Xiong P (2024) Optimization strategies for CO_2_ biological fixation. Biotechnol Adv 73:108364. 10.1016/j.biotechadv.2024.108364 38642673 10.1016/j.biotechadv.2024.108364

[CR67] Liu X, Luo H, Yu D, Tan J, Yuan J, Li H (2022) Synthetic biology promotes CO_2_ capture to produce fatty-acid derivatives in microbial cell factories. Bioresour Bioproc 9:124. 10.1186/s40643-022-00615-2 10.1186/s40643-022-00615-2 PMC10992411 38647643

[CR68] Ljungdahl LG (1986) The autotrophic pathway of acetate synthesis in acetogenic bacteria. Annu Rev Microbiol 40:415–450. 10.1146/annurev.mi.40.100186.002215 3096193 10.1146/annurev.mi.40.100186.002215

[CR69] Maheshwari N, Kumar M, Thakur IS, Srivastava S (2017) Recycling of carbon dioxide by free air CO_2_ enriched (FACE) *Bacillus* sp. SS105 for enhanced production and optimization of biosurfactant. Bioresour Technol 242:2–6. 10.1016/j.biortech.2017.03.124 28372863 10.1016/j.biortech.2017.03.124

[CR70] Maheshwari N, Thakur IS, Srivastava S (2022) Role of carbon-dioxide sequestering bacteria for clean air and prospective production of biomaterials: a sustainable approach. Environ Sci Pollut Res 29. 10.1007/s11356-022-19393-7 10.1007/s11356-022-19393-7 35304714

[CR71] Markande AR, Patel D, Varjani S (2021) Biosurfactants: properties, applications and current developments. Bioresour Technol 330:124963. 10.1016/j.biortech.2021.124963 33744735 10.1016/j.biortech.2021.124963

[CR72] Mills LA, McCormick AJ, Lea-Smith DJ (2020) Current knowledge and recent advances in understanding metabolism of the model cyanobacterium *Synechocystis* sp. PCC 6803. Biosci Rep. 10.1042/BSR20193325 10.1042/BSR20193325 PMC7133116 32149336

[CR73] Mishra S, Raghuvanshi S, Gupta S, Raj K (2017) Application of novel thermo-tolerant haloalkalophilic bacterium *Halomonas stevensii* for bio-mitigation of gaseous CO_2_: energy assessment and product evaluation. Process Biochem 55:133–145. 10.1016/j.procbio.2017.01.019

[CR74] Mistry AN, Ganta U, Chakrabarty J, Dutta S (2019) Biological systems for CO_2_ sequestration: organisms and their pathways—a review. Environ Prog Sustain Energy 38:127–136. 10.1002/ep.12946

[CR75] Mohapatra RK, Padhi D, Sen R, Nayak M (2022) Bio-inspired CO₂ capture and utilization by microalgae for bioenergy feedstock production: a greener approach for environmental protection. Bioresour Technol Rep 19:101116. 10.1016/j.biteb.2022.101116

[CR76] Moro GV, Almeida RTR, Napp AP, Porto C, Pilau EJ, Lüdtke DS, Moro AV, Vainstein MH (2018) Identification and UHPLC-HRMS characterization of biosurfactants, including a new surfactin, from oil-contaminated environments. Microb Biotechnol 11:759–769. 10.1111/1751-7915.13276 29761667 10.1111/1751-7915.13276 PMC6011949

[CR77] Napp AP, Pereira JES, Oliveira JS, Silva-Portela RCB, Agnez-Lima LF, Peralba MCR, Bento FM, Passaglia LMP, Thompson CE, Vainstein MH (2018) Comparative metagenomics reveals different hydrocarbon degradative abilities from enriched oil-drilling waste. Chemosphere 209:7–16. 10.1016/j.chemosphere.2018.06.068 29908430 10.1016/j.chemosphere.2018.06.068

[CR78] Nguyen E, Poli M, Durrant MG, Kang B, Katrekar D, Li DB, Bartie LJ, Thomas AW, King SH, Brixi G, Sullivan J, Ng MY, Lewis A, Lou A, Ermon S, Baccus SA, Hernandez-Boussard T, Ré C, Hsu PD, Hie BL (2024) Sequence modeling and design from molecular to genome scale with Evo. Science 386. 10.1126/science.ado9336 10.1126/science.ado9336 PMC12057570 39541441

[CR79] Nihorimbere V, Cawoy H, Seyer A, Brunelle A, Thonart P, Ongena M (2012) Impact of rhizosphere factors on cyclic lipopeptide signature from the plant-beneficial strain *Bacillus amyloliquefaciens* S499. FEMS Microbiol Ecol 79:176–191. 10.1111/j.1574-6941.2011.01208.x 22029651 10.1111/j.1574-6941.2011.01208.x

[CR80] Nozawa T, Tanikawa T, Hasegawa H, Takahashi C, Ando Y, Matsushita M, Nakagawa Y, Matsuyama T (2007) Rhamnolipid-dependent spreading growth of *Pseudomonas aeruginosa* on high-agar medium: enhancement under CO_2_-rich anaerobic conditions. Microbiol Immunol 51:703–712. 10.1111/j.1348-0421.2007.tb03959.x 17704632 10.1111/j.1348-0421.2007.tb03959.x

[CR81] Occhipinti PS, Russo N, Foti P, Zingale IM, Pino A, Romeo FV, Randazzo CL, Caggia C (2024) Current challenges of microalgae applications: exploiting the potential of non-conventional species. J Sci Food Agric 104:3823–3833. 10.1002/jsfa.13136 37971887 10.1002/jsfa.13136

[CR82] Oh S, Greene J, Honegger M, Michaelowa A (2025) Review of economics and policies of carbon dioxide removal. Curr Sustain Renew Energy Rep 12. 10.1007/s40518-025-00252-1 10.1007/s40518-025-00252-1 PMC11905288 40083478

[CR83] Park T, Joo HW, Kim GY, Kim S, Yoon S, Kwon TH (2017) Biosurfactant as an enhancer of geologic carbon storage: microbial modification of interfacial tension and contact angle in CO_2_/water/quartz systems. Front Microbiol 8:1285. 10.3389/fmicb.2017.01285 28744272 10.3389/fmicb.2017.01285 PMC5504122

[CR84] Phetcharat T, Dawkrajai P, Chitov T, Mhuantong W, Champreda V, Bovonsombut S (2019) Biosurfactant-producing capability and prediction of functional genes beneficial to MEOR in indigenous bacterial communities of an onshore oil reservoir. Curr Microbiol 76:382–391. 10.1007/s00284-019-01641-8 30734843 10.1007/s00284-019-01641-8

[CR85] Pinzon NM, Cook AG, Ju L-K (2013) Continuous rhamnolipid production using denitrifying *Pseudomonas aeruginosa* cells in a hollow-fiber bioreactor. Biotechnol Prog 29:352–358. 10.1002/btpr.1701 23359613 10.1002/btpr.1701

[CR86] Qin WQ, Liu YF, Gang HZ, Liu JF, Zhou L, Yang SZ, Mu BZ (2025) Structural diversity of surfactin lipopeptides and their molecular behaviors at interfaces. Adv Colloid Interface Sci 343:103581. 10.1016/j.cis.2025.103581 40570658 10.1016/j.cis.2025.103581

[CR87] Rajkumar R, Takriff MS, Veeramuthu A (2022) Technical insights into CO_2_ sequestration by microalgae: a biorefinery approach. Biomass Convers Biorefin 12. 10.1007/s13399-022-02446-9

[CR88] Rajput SD, Keshavkant S (2025) V sequestration and single-cell protein production: opportunities and challenges in a circular economy. Chem Eng J 515:163585. 10.1016/j.cej.2025.163585

[CR89] Razzak S, Bahar K, Islam KMO, Haniffa AK, Faruque MO, Hossain SMZ, Hossain MM (2024) Microalgae cultivation in photobioreactors: sustainable solutions for a greener future. Green Chemical Engineering 5(4):418–439. 10.1016/j.gce.2023.10.004

[CR90] Rising J, Tedesco M, Piontek F, Stainforth DA (2022) The missing risks of climate change. Nature 610:643–651. 10.1038/s41586-022-05243-6 36289386 10.1038/s41586-022-05243-6

[CR91] Ritchie H, Rosado P, Roser M (2023) CO2 and greenhouse gas emissions. Our World in Data. https://ourworldindata.org/co2-and-greenhouse-gas-emissions. Accessed 18 Nov 2025

[CR92] Ruan S, Jiang Y, Wang A, Zhang X, Lin Y, Liang S (2025) Carbon sequestration pathways in microorganisms: advances, strategies and applications. Eng Microbiol 5:100196. 10.1016/j.engmic.2025.100196 41982409 10.1016/j.engmic.2025.100196 PMC12967845

[CR93] Santos D, Napp AP, Aguiar CP, Dutra WL, Gonçalves BX, Vecchia D, Tewari RDB (2024) Carbon dioxide as a carbon source for biosurfactant production. SSRN preprint 5066224. https://ssrn.com/abstract=5066224

[CR94] Sarubbo LA, Silva MGC, Durval IJB, Bezerra KGO, Ribeiro BG, Silva IA, Twigg MS, Banat IM (2022) Biosurfactants: production, properties, applications, trends, and perspectives. Biochem Eng J 181:108377. 10.1016/j.bej.2022.108377

[CR95] Sasse A, Chikina M, Mostafavi S (2024) Unlocking gene regulation with sequence-to-function models. Nat Methods 21:1374–1377. 10.1038/s41592-024-02331-5 39122947 10.1038/s41592-024-02331-5

[CR96] Sharma N, Ahlawat YK, Sharma AJ, Chamoli N, Thakur M, Sharma A, Mehmood S, Malik A, Ahmed M, Punia H, Choubey S (2025) A comprehensive review on microbial production and significant applications of multifunctional biomolecules: biosurfactants. Biodegradation 36(2):26. 10.1007/s10532-025-10121-9 10.1007/s10532-025-10121-9 40159571

[CR97] Simon E, Swanson K, Zou J (2024) Language models for biological research: a primer. Nat Methods 21:1422–1429. 10.1038/s41592-024-02354-y 39122951 10.1038/s41592-024-02354-y

[CR98] Strauss G, Fuchs G (1993) Enzymes of a novel autotrophic CO_2_ fixation pathway in *Chloroflexus aurantiacus*, the 3-hydroxypropionate cycle. Eur J Biochem 215:633–643. 10.1111/j.1432-1033.1993.tb18074.x 8354269 10.1111/j.1432-1033.1993.tb18074.x

[CR99] Sultan F, Maji D, Phatake RS, Kumar K (2025) Pharmaceutical applications of microbial biosurfactants. Int J Pharm 681:125887. 10.1016/j.ijpharm.2025.125887 40555356 10.1016/j.ijpharm.2025.125887

[CR100] Sundaram S, Thakur IS (2015) Biosurfactant production by a CO_2_-sequestering *Bacillus* sp. strain ISTS2. Bioresour Technol 188:247–250. 10.1016/j.biortech.2015.01.029 25641713 10.1016/j.biortech.2015.01.029

[CR101] Thiedemann TM, Wark M (2025) A compact review of current technologies for carbon capture as well as storing and utilizing captured CO2. Processes 13. 10.3390/pr13010283

[CR102] Tollefson J (2021) COP26 climate summit: a scientists’ guide to a momentous meeting. Nature 599:15–17 34697485 10.1038/d41586-021-02815-w

[CR103] Wahby BE, Lay CH, Lin CY (2025) Anaerobic enrichment of bio-surfactant-producing bacteria from oil-polluted marine soils using mixed carbon sources. Bioresource Technol Rep. 10.1016/j.biteb.2025.102425

[CR104] Wenjie X, Li Y, Ping W, Jianlong X, Hanping D (2012) Characterization of a thermophilic and halotolerant Geobacillus pallidus H9 and its application in microbial enhanced oil recovery (MEOR). Ann Microbiol 62(4):1779–1789. 10.1007/s13213-012-0436-5

[CR105] Widberger J, Wittgens A, Klaunig S, Krämer M, Kissmann AK, Höfele F, Baur T, Weil T, Henkel M, Hausmann R, Bengelsdorf FR, Eikmanns BJ, Dürre P, Rosenau F (2024) Recombinant production of *Pseudomonas aeruginosa* rhamnolipids in *P. putida* KT2440 on *Acetobacterium woodii* cultures grown chemo-autotrophically with carbon dioxide and hydrogen. Microorganisms. 10.3390/microorganisms12030529 38543580 10.3390/microorganisms12030529 PMC10972445

[CR106] Willenbacher J, Rau JT, Rogalla J, Syldatk C, Hausmann R (2015) Foam-free production of Surfactin via anaerobic fermentation of Bacillus subtilis DSM 10T. AMB Express 5(1). 10.1186/s13568-015-0107-6 10.1186/s13568-015-0107-6 PMC4385232 25852998

[CR107] Yakimov MM, Amro MM, Bock M, Boseker K, Fredrickson HL, Kessel DG, Timmis KN (1997) The potential of *Bacillus licheniformis* strains for in situ enhanced oil recovery. J Pet Sci Eng 18:147–160. 10.1016/S0920-4105(97)00015-6

[CR108] Yakimov MM, Timmis KN, Wray V, Fredrickson HL (1995) Characterization of a new lipopeptide surfactant produced by thermotolerant and halotolerant subsurface *Bacillus licheniformis* BAS50. Appl Environ Microbiol 61:1706–1713. 10.1128/AEM.61.5.1706-1713.1995 10.1128/aem.61.5.1706-1713.1995 PMC167432 7646007

[CR109] Yan X, He Q, Geng B, Yang S (2024) Microbial cell factories in the bioeconomy era: from discovery to creation. BioDesign Res 6:0052. 10.34133/bdr.0052 10.34133/bdr.0052 PMC11491672 39434802

[CR110] Yen TF, Park JK, Lee KI, Li Y (1991) Fate of surfactant vesicles surviving from thermophilic, halotolerant, spore-forming *Clostridium thermohydrosulfuricum*. Dev Pet Sci 31:C297–C309. 10.1016/S0376-7361(09)70167-0

[CR111] Yousaf M, Zaman M, Mahmood A, Imran M, Elkamel A, Rizwan M, Wilberforce T, Riaz F (2022) Carbon dioxide utilization: a critical review from a multiscale perspective. Energy Sci Eng 10:4890–4923. 10.1002/ese3.1303

[CR112] Youssef N, Simpson DR, Duncan KE, McInerney MJ, Folmsbee M, Fincher T, Knapp RM (2007) In situ biosurfactant production by *Bacillus* strains injected into a limestone petroleum reservoir. Appl Environ Microbiol 73(4):1239–1247. 10.1128/AEM.02264-06 17172458 10.1128/AEM.02264-06 PMC1828672

[CR113] Youssef NH, Duncan KE, McInerney MJ (2005) Importance of 3-hydroxy fatty-acid composition of lipopeptides for biosurfactant activity. Appl Environ Microbiol 71:7690–7695. 10.1128/AEM.71.12.7690-7695.2005 16332741 10.1128/AEM.71.12.7690-7695.2005 PMC1317328

[CR114] Yu H-Y, Wang S-G, Xia P-F (2022) Reprogramming microbial CO_2_-metabolizing chassis with CRISPR-Cas systems. Front Bioeng Biotechnol 10:897204. 10.3389/fbioe.2022.897204 35814004 10.3389/fbioe.2022.897204 PMC9260013

[CR115] Zhang R-S, Wang F-G, Qi Z-Q, Qiao J-Q, Du Y, Yu J-J, Yu M-N, Liang D, Song T-Q, Yan P-X, Cao H-J, Zhang H, Liu Y-F (2022) Iturins produced by *Bacillus velezensis* Jt84 play a key role in biocontrol of rice blast disease. Biol Control 174:105001. 10.1016/j.biocontrol.2022.105001

[CR116] Zhao F, Cui Q, Han S, Dong H, Zhang J, Ma F, Zhang Y (2015) Enhanced rhamnolipid production of *Pseudomonas aeruginosa* SG by increasing copy number of rhlAB genes with modified promoter. RSC Adv 5:70546–70552. 10.1039/c5ra13415c

[CR117] Zhao F, Li P, Guo C, Shi R-J, Zhang Y (2018) Bioaugmentation of oil reservoir indigenous *Pseudomonas aeruginosa* to enhance oil recovery through in-situ biosurfactant production without air injection. Bioresour Technol 251:295–302. 10.1016/j.biortech.2017.12.057 29289873 10.1016/j.biortech.2017.12.057

[CR118] Zhao F, Ma F, Shi R, Zhang J, Han S, Zhang Y (2015) Production of rhamnolipids by *Pseudomonas aeruginosa* is inhibited by H2S but resumes in co-culture with *P. stutzeri*: applications for MEOR. Biotechnol Lett 37:1803–1808. 10.1007/s10529-015-1859-4 25994582 10.1007/s10529-015-1859-4

[CR119] Zhao F, Mandlaa M, Hao J, Liang X, Shi R, Han S, Zhang Y (2014) Optimization of culture medium for anaerobic production of rhamnolipid by recombinant *Pseudomonas stutzeri* Rhl for MEOR. Lett Appl Microbiol 59:231–237. 10.1111/lam.12269 24738996 10.1111/lam.12269

[CR120] Zhao F, Wang Q, Zhang Y, Lei L (2021b) Anaerobic biosynthesis of rhamnolipids by *Pseudomonas aeruginosa*: performance, mechanism and potential for enhanced oil recovery. Microb Cell Fact 20. 10.1186/s12934-021-01593-4 10.1186/s12934-021-01593-4 PMC8139158 34016105

[CR121] Zhao F, Wang Y, Hu X, Huang X (2022) Simple and efficient screening of microbial strains capable of anaerobic biosynthesis of biosurfactants: method, factors and application evaluation. Front Microbiol 13:989998 36171744 10.3389/fmicb.2022.989998 PMC9511215

[CR122] Zhao F, Wu Y, Liu L (2025) Enhanced anaerobic synthesis of rhamnolipids and emulsification of crude oil by genetically engineered Pseudomonas aeruginosa strains. Microb Cell Fact 24(1). 10.1186/s12934-025-02793-y 10.1186/s12934-025-02793-y PMC12265193 40665288

[CR123] Zhao F, Zhang J, Shi R, Han S, Ma F, Zhang Y (2015) Production of biosurfactant by a *Pseudomonas aeruginosa* isolate and its applicability to in situ MEOR under anoxic conditions. RSC Adv 5:36044–36050. 10.1039/c5ra03559g

[CR124] Zhao F, Zhu H, Cui Q, Wang B, Su H, Zhang Y (2021) Anaerobic production of surfactin by a new *Bacillus subtilis* isolate and in situ emulsification/viscosity reduction toward EOR applications. J Pet Sci Eng 201:108508. 10.1016/j.petrol.2021.108508

[CR125] Zheng C, Yu L, Huang L, Xiu J, Huang Z (2012) Investigation of a hydrocarbon-degrading strain, *Rhodococcus ruber* Z25, for potential MEOR. J Pet Sci Eng 81:49–56. 10.1016/j.petrol.2011.12.019

[CR126] Zhou X, Li L, Sun S, Xiong P, Liu X (2025) Production of succinate with additional CO_2_-fixation reactions facilitated by RuBisCO-based engineered *Escherichia coli*. Biotechnol Prog 41:e70015. 10.1002/btpr.70015 39968675 10.1002/btpr.70015

[CR127] Zhou Y, Cui X, Wu B, Wang Z, Liu Y, Ren T, Xia S, Rittmann BE (2024) Microalgal extracellular polymeric substances (EPS) and their roles in cultivation, biomass harvesting, and bioproducts extraction. Bioresour Technol 406:131054. 10.1016/j.biortech.2024.131054 38944317 10.1016/j.biortech.2024.131054

